# Interplay Between Polymorphic Short Tandem Repeats and Gene Expression Variation in *Caenorhabditis elegans*

**DOI:** 10.1093/molbev/msad067

**Published:** 2023-03-31

**Authors:** Gaotian Zhang, Erik C Andersen

**Affiliations:** Department of Molecular Biosciences, Northwestern University, Evanston, IL; Department of Molecular Biosciences, Northwestern University, Evanston, IL

**Keywords:** short tandem repeats, gene regulation, expression QTL, oxidative stress, antioxidant genes, *Caenorhabditis elegans*

## Abstract

Short tandem repeats (STRs) have orders of magnitude higher mutation rates than single nucleotide variants (SNVs) and have been proposed to accelerate evolution in many organisms. However, only few studies have addressed the impact of STR variation on phenotypic variation at both the organismal and molecular levels. Potential driving forces underlying the high mutation rates of STRs also remain largely unknown. Here, we leverage the recently generated expression and STR variation data among wild *Caenorhabditis elegans* strains to conduct a genome-wide analysis of how STRs affect gene expression variation. We identify thousands of expression STRs (eSTRs) showing regulatory effects and demonstrate that they explain missing heritability beyond SNV-based expression quantitative trait loci. We illustrate specific regulatory mechanisms such as how eSTRs affect splicing sites and alternative splicing efficiency. We also show that differential expression of antioxidant genes and oxidative stresses might affect STR mutations systematically using both wild strains and mutation accumulation lines. Overall, we reveal the interplay between STRs and gene expression variation by providing novel insights into regulatory mechanisms of STRs and highlighting that oxidative stress could lead to higher STR mutation rates.

## Introduction

Genetic variation can cause significant differences in gene expression among individuals. Mutations in regulatory elements, such as promoters and enhancers, might only affect the expression of single genes, whereas mutations altering structures and abundances of diffusible factors, such as transcription factors (TFs) and chromatin cofactors, might affect the expression of multiple genes across the genome. Quantitative genetic mapping techniques, including both linkage and genome-wide association (GWA) mapping studies, enable the identification of genome-wide variants that influence gene expression and other complex traits. A genomic locus that contains alleles showing significant association with mRNA expression variation is called an expression quantitative trait locus (eQTL) (Brem et al. 2002; West et al. 2007; Albert et al. 2018; GTEx Consortium 2020; Zhang, Roberto, et al. 2022). Although thousands of eQTL have been detected in different organisms, associated genetic variants are mostly limited to single nucleotide variants (SNVs) and short insertions or deletions (indels) (Brem et al. 2002; West et al. 2007; Rockman et al. 2010; Zan et al. 2016; GTEx Consortium et al. 2017, 2020; Kita et al. 2017; Albert et al. 2018; Evans and Andersen 2020; Snoek et al. 2021; Zhang, Roberto, et al. 2022). Emerging studies successfully linked gene expression variation to other types of DNA variants, such as short tandem repeats (STRs) and structural variants (Boettger et al. 2016; Gymrek et al. 2016; Sekar et al. 2016; Press et al. 2018; Song et al. 2018; Fotsing et al. 2019; Jakubosky et al. 2020; Reinar et al. 2021).

STRs are repetitive elements consisting of 1–6 bp DNA sequence motifs (Willems et al. 2016; Fotsing et al. 2019). Compared to SNVs and short indels, STR mutations show 1) orders of magnitude higher mutation rates (Lynch 2010; Sun et al. 2012; Willems et al. 2016; Gymrek et al. 2017), 2) higher incidence of insertions or deletions, mostly in the number of repeats (Mirkin 2007; Gemayel et al. 2010), 3) more multiallelic sites (Gymrek 2017), and 4) more de novo mutations (Willems et al. 2016; Gymrek 2017). Dozens of human diseases have been associated with STR mutations (Mirkin 2007). Various effects of STR variation on regulation of gene expression have also been suggested from both in vitro and in vivo studies across a wide range of taxa (Weiser et al. 1989; Rothenburg et al. 2001; Contente et al. 2002; Rockman and Wray 2002; Sureshkumar et al. 2009; Vinces et al. 2009; Yáñez-Cuna et al. 2014). However, these STRs only represented a small fraction of STRs in genomes. To our best knowledge, systematic evaluation of GWAs between STR variation and gene expression variation have only been applied in humans (Gymrek et al. 2016; Quilez et al. 2016; Fotsing et al. 2019) and *Arabidopsis thaliana* (Press et al. 2018; Reinar et al. 2021), in part because of the difficulties in accurately genotyping STRs throughout the genome in large scales (Willems et al. 2017).

We have recently studied the natural variation in gene expression (Zhang, Roberto, et al. 2022) and STRs (Zhang, Wang, et al. 2022) across wild strains of the nematode *Caenorhabditis elegans*. We collected reliable expression measurements for 25,849 transcripts of 16,094 genes in 207 *C. elegans* strains using bulk mRNA sequencing and identified 6,545 eQTL underlying expression variation of 5,291 transcripts of 4,520 genes using GWA mappings (Zhang, Roberto, et al. 2022). We characterized 9,691 polymorphic STRs (pSTRs) with motif lengths of 1–6 bp across the species, including the 207 strains above, using high-throughput genome sequencing data (Zhang, Wang, et al. 2022) and a bioinformatic tool previously demonstrated to be reliable for large-scale profiling of STRs (Gymrek et al. 2016; Willems et al. 2017).

In this work, we leveraged the recently generated expression (Zhang, Roberto, et al. 2022) and STR (Zhang, Wang, et al. 2022) data from 207 wild *C. elegans* strains to conduct a genome-wide scan of how STRs affect gene expression variation. We identified 3,118 and 1,857 expression STRs (eSTRs) that were associated with expression of nearby and remote genes, respectively. We found that eSTRs might help explain missing heritability in SNV-based eQTL studies for both local and distant eQTL. We also explored specific mechanisms of eSTRs and illustrated how local eSTRs might have influenced alternative splicing sites to cause differential transcript usage. We showed that expression of several genes in the same pathway might be altered because of a distant eSTR in a gene upstream. We also found evidence that expression variation in an antioxidant gene, *ctl-1*, might underlie STR variation across wild *C. elegans* strains. We further determined the positive relationship between endogenous oxidative stress and STR insertions/deletions using three mutation accumulation (MA) line panels. Our results demonstrate the systemic influences of eSTRs on gene expression and the potential effects of expression variation in antioxidant genes on STR mutations in *C. elegans*. We reveal the interplay between STRs and gene expression variation and provide publicly available frameworks to associate STRs with variation in gene expression and other complex traits in future studies.

## Results

### Variation in STRs Regulates Expression in Nearby Genes

**Fig. 1.**
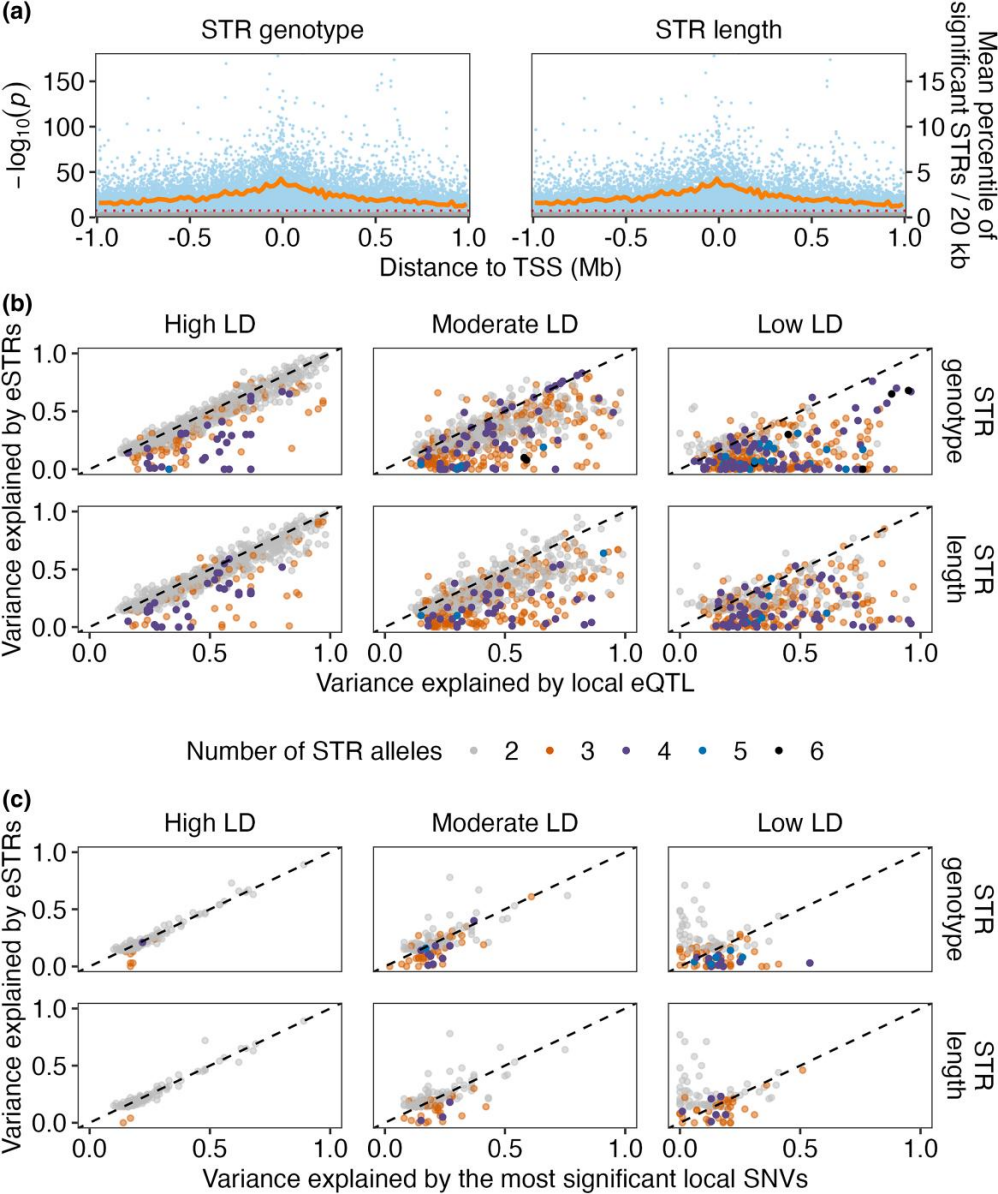
eSTRs identified using LRTs. (*a*) Identification of eSTRs using LRTs on full (including STR variation as a variable) and reduced (excluding STR variation as a variable) models. The effects of STR variation in genotype (left panel) or length (right panel) were analyzed separately as factorial variables. Each dot represents a test between STR and transcript expression variation and is plotted with the distance of the STR to the transcription start site (TSS) of the transcript (*x*-axis) against its −log10 (*P*) value (*y*-axis on the left). Blue and gray dots represent tests using real and permuted data of STR variation, respectively. The red dotted horizontal lines represent Bonferroni thresholds. The dark orange lines represent the mean percentage of significant test (real data) above the Bonferroni thresholds in each 20 kb bin (*y*-axis on the right). (*b*) The VE by local eQTL that were identified using GWA mapping experiments (Zhang, Roberto, et al. 2022) was plotted against the VE for the most significant eSTRs. (*c*) The VE by the TopSNVs was plotted against the VE of the most significant eSTRs. Dots are colored by the number of STR alleles used in eSTR VE calculation. LD (*r*^2^) between eQTL and eSTRs were used to separate panels on the *x*-axis, with high LD (*r*^2^ ≥ 0.7), moderate LD (0.3 ≤ *r*^2^ < 0.7), and low LD (*r*^2^ < 0.3). The dashed lines on the diagonal are shown as visual guides to represent VE_eQTL/TopSNVs_ = VE_eSTRs_.

We obtained expression data of 25,849 transcripts (Zhang, Roberto, et al. 2022) of 16,094 genes and 9,691 pSTRs (Zhang, Wang, et al. 2022) across 207 wild *C. elegans* strains. We investigated the effects of pSTRs on transcript expression of nearby genes using a likelihood-ratio test (LRT) to evaluate the association between STR variation and transcript expression variation for all pSTRs within 2 Mb surrounding each transcript and with at least two common alleles (allele frequency > 0.05) (supplementary fig. S1*a*, Supplementary Material online). We applied the LRT using both pSTR genotypes and lengths by treating them as factorial variables (see Materials and Methods). In total, using STR genotypes, 1,555,828 tests were performed to test the effect of 3,335 pSTRs on the expression variation of 25,849 transcripts, each of which was tested for a median of 59 STRs (ranging from 1 to 141) (fig. 1*a* and supplementary fig. S2, Supplementary Material online). Using STR lengths, 1,227,485 tests were performed for the effect of 2,607 pSTRs on the expression variation of 25,847 transcripts, each of which was tested for a median of 47 STRs (ranging from 1 to 119) (fig. 1*a* and supplementary fig. S2, Supplementary Material online). For each test, we also performed another test using permuted STR genotypes or lengths. We identified local eSTRs with LRT values that passed the Bonferroni threshold (3.2E-8 and 4.1E-8 for STR genotypes and lengths, respectively) and found 3,082 eSTRs for 2,888 transcripts by STR genotypes and 2,391 eSTRs for 2,791 transcripts by STR lengths, including 2,355 eSTRs for 2,695 transcripts by both STR genotypes or lengths (fig. 1*a* and supplementary fig. S2 and data S1, Supplementary Material online). Each transcript had a median of nine eSTRs (ranging from 1 to 77) and six eSTRs (ranging from 1 to 65) by STR genotypes and lengths, respectively. None of the tests using permuted STRs passed the Bonferroni thresholds (fig. 1*a* and supplementary data S1, Supplementary Material online). As expected, we observed that STRs in close proximity to or within a transcript were more likely to pass the significance threshold than STRs far away from the transcript (fig. 1*a* and supplementary fig. S2, Supplementary Material online), indicating a close relationship between STRs and gene expression.

In our recent eQTL study (Zhang, Roberto, et al. 2022), we classified eQTL into local eQTL (located close to the genes that they influence) and distant eQTL (located farther away from the genes that they influence) (supplementary fig. S1*b*, Supplementary Material online). Among the 3,185 transcripts with local eQTL (Zhang, Roberto, et al. 2022), 2,477 were also found with eSTRs (enrichment tested by one-sided Fisher's exact test, with *P* = 2.2E-16). To compare the effects of eQTL and eSTRs in gene regulation, we compared the expression variance explained (VE) by eQTL and the most significant eSTR for each transcript and the linkage disequilibrium (LD) between them (fig. 1*b*). Most eQTL-eSTR pairs (48%) with high LD (*r^2^* ≥ 0.7) explained similar levels of expression variance (fig. 1*b*), suggesting that these eSTRs might be detected because of the high LD to eQTL or vice versa. More than half of the eQTL-eSTR pairs showed moderate LD (0.3 ≤ *r^2^* < 0.7, 35%) or low LD (*r^2^* < 0.3, 17%), suggesting that they might be independent from each other (fig. 1*b*). Generally, multiallelic eSTRs explained less variance than eQTL (fig. 1*b*). The effects of a multiallelic STR could be underestimated if some of its alleles affected expression, whereas other alleles only added noise to the estimation. Furthermore, allele frequencies of different alleles in a multiallelic STR could also affect the estimation of VE. Under any circumstances, eSTRs might help explain more variance in expression than the sole contribution from eQTL. Additionally, 482 transcript expression traits were detected with eSTRs but not eQTL (fig. 1*c*). To compare these unique eSTRs with nearby SNVs, we selected SNVs (TopSNVs) that were within 2 Mb surrounding each of the 482 transcripts and were the most significant markers in the previous GWA mapping experiments (Zhang, Roberto, et al. 2022). Then, we calculated the VE by the eSTRs and the TopSNVs in transcript expression variation and their LD (fig. 1*c*). Among the 876 eSTR-TopSNV pairs, eSTRs explained more expression variance than the TopSNVs in 392 pairs (45% in 876), with 90, 145, and 157 in high, moderate, and low LD pairs, respectively (fig. 1*c*). Altogether, these results further suggested the independent effects of eSTR on expression abundance in some transcripts. To further evaluate if eSTRs might help explain missing heritability (Hannan 2010), we estimated narrow-sense heritability (*h^2^*) using only SNV genotype data or a combination of both SNV and STR data for each of the 25,849 transcript expression traits (see Materials and Methods; supplementary fig. S3, Supplementary Material online). We observed increased *h^2^* estimation in 18,658 traits (72%) (supplementary fig. S3, Supplementary Material online), showing that STR increased heritability estimation in the majority of expression traits and suggesting that STR could help explain missing heritability from estimation using SNVs.

### Insertion in a Local eSTR Affects Transcript Isoform Usage

**Fig. 2.**
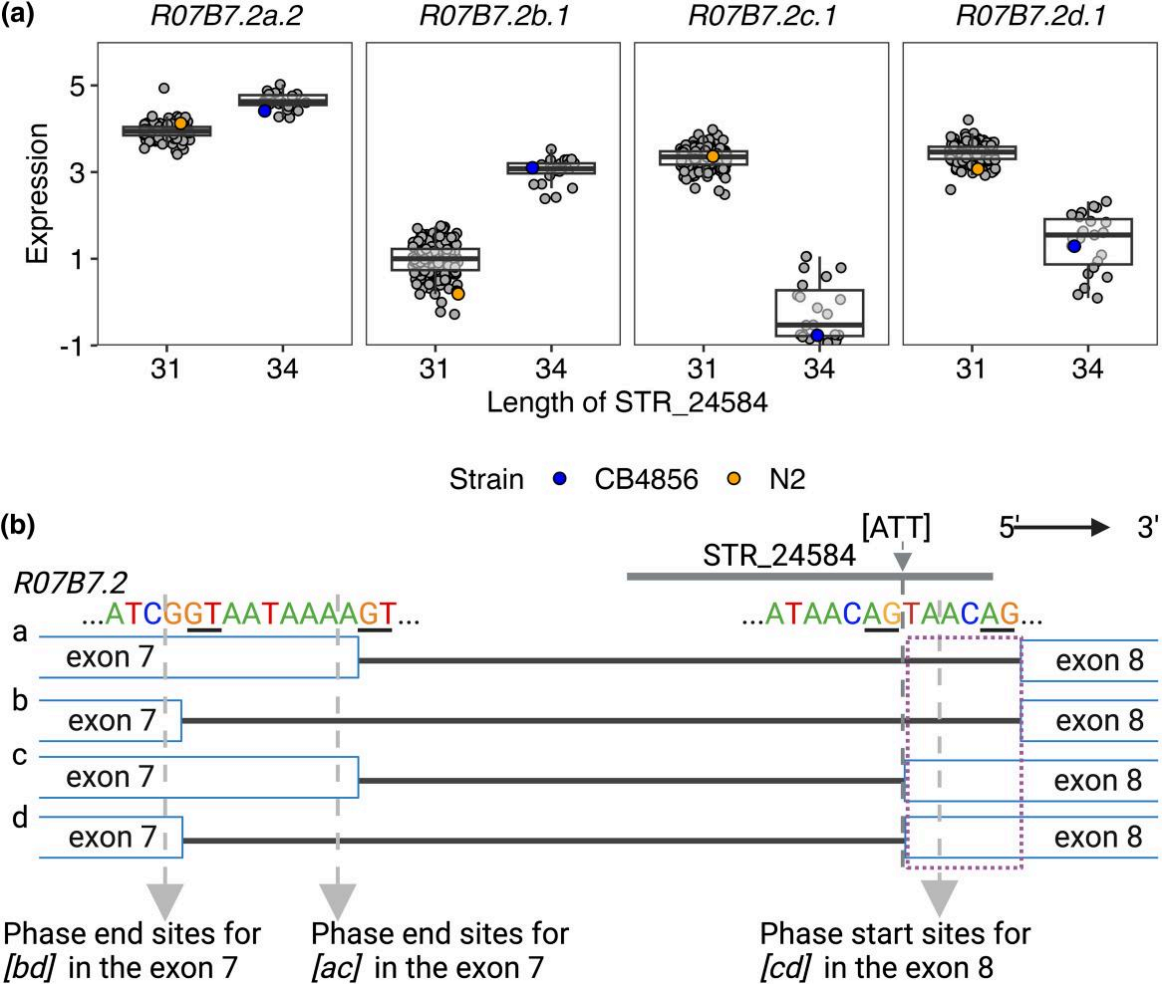
eSTRs disrupting splicing. (*a*) Tukey box plots showing expression variation of four transcripts of the gene *R07B7.2* between strains with different lengths of the STR_24584. Each point corresponds to a strain. The reference strain N2 and the wild strain CB486 are colored orange and blue, respectively. Other strains are colored gray. Box edges denote the 25th and 75th quantiles of the data; and whiskers represent 1.5× the interquartile range. (*b*) Graphic illustration of sequences in the splice site of four transcripts of the gene *R07B7.2* and the position of STR_24584. The dashed arrow in dark gray indicates the position of a 3-bp insertion in the STR_24584 and the splicing region of *R07b7.2[ab]*. The dashed arrow in light gray indicates the phase start and end sites for different exons. The purple dashed rectangle indicates the genomic region from 12,057,480 to 12,057,485 bp on the chromosome V. Created using BioRender.com.

We next focused on eSTRs that were in genomic features (coding regions [CDS], 5′ untranslated region [UTR], 3′ UTR, promoter, enhancer, and intron) of their target transcripts and were outside of hyper-divergent regions (Lee et al. 2021). We predicted the functional consequences (Li 2011) of these eSTRs and found a total of 13 eSTRs in 16 transcripts of 12 genes that showed high-impact mutations, including missense mutations, in-frame insertions and deletions, start lost, stop gain, and mutations in splicing regions or acceptors. Another 17 eSTRs in 21 transcripts of 17 genes were predicted to affect 5′ UTRs and 3′ UTRs. We identified two enriched motif sequences, ATTTTT and ATGTT, in these eSTRs by STR genotypes (one-sided Fisher exact test, Bonferroni-corrected *P* = 0.04 and 6.8E-5, respectively) or STR lengths (one-sided Fisher exact test, Bonferroni-corrected *P* = 0.03 and 4.6E-5, respectively). Instead of finding multiple eSTRs, the two motif sequences only came from two eSTRs, STR_13795 of (ATTTTT)_5_ and STR_24584 of (ATGTT)_6.2_, each of which was associated with multiple transcripts of the same genes. In particular, STR_24584 was predicted to have high-impact mutations in the splicing regions of four transcripts of the gene, *R07B7.2*, and was associated with their expression variation (fig. 2). Compared to strains with the reference allele, strains with a 3-bp insertion showed significantly higher expression in the isoforms *R07B7.2[ab]* but significantly lower expression in the isoforms *R07B7.2[cd]* (fig. 2*a*). More specifically, the insertion was located at the 3′ splice site in the intron between exon 7 and exon 8 of *R07B7.2[ab]* and at the junction of the intron and exon 8 for *R07B7.2[cd]* (fig. 2*b*).

To confirm the expression quantification of the four *R07B7.2* transcripts were relatively accurate and their differential expression among wild strains was not biased by our methods, we performed the following analyses on the six replicates of the reference strain N2 and another commonly used strain CB4856 (fig. 2*a*). First, we examined their RNA sequencing alignment in Binary Alignment Map (BAM) files which were pseudo-mapped using *Kallisto* (Bray et al. 2016; Zhang, Roberto, et al. 2022). We focused on the 6 bp in the above 3′ splice site (12,057,480 to 12,057,485 bp on the chromosome V) and the closeby 6 bp (12,057,474 to 12,057,479 bp) (fig. 2*b* and supplementary fig. S4, Supplementary Material online). In the three replicates of N2, the numbers of reads mapped to the 3′ splice site were about half of those in the closeby regions, which might correspond to two (*R07B7.2[cd]*) of the four transcripts that have the elongated exon 8 (fig. 2*b* and supplementary fig. S4 and table S1, Supplementary Material online). In the three replicates of the CB4856 strain, however, the numbers of reads mapped to the 3′ splice site were about 10% of those in the closeby regions, indicating lower expression of *R07B7.2[cd]* than *R07B7.2[ab]* in the CB4856 strain (supplementary fig. S4 and table S1, Supplementary Material online). Second, we performed a differential exon usage (DEU) analysis between the CB4856 and N2 strains with a prior real alignment using *STAR* (Dobin et al. 2013) (see Materials and Methods). We detected significant DEU in the 3′ splice site between the CB4856 and N2 strains (supplementary fig. S5 and data S2, Supplementary Material online). Altogether, these results confirmed possible differential alternative splicing events in *R07B7.2* between the CB4856 and N2 strains.

Next, we attempted to dissect how STR variation might have affected alternative splicing. We speculated that at least two mechanisms might underlie the expression differences among the four transcripts caused by STR_24584 variation. First, the insertion [ATT] changed the 3′ splice site of *R07B7.2[ab]* from 5′-GTAACAG-3′ to 5′-TTAACAG-3′ (fig. 2*b*), which became closer to the conserved consensus sequence 5′-UUUUCAG-3′ of the 3′ splice site in *C. elegans* (Blumenthal and Steward 2011). Therefore, the insertion might promote splicing efficiency for *R07B7.2[ab]* in pre-mRNAs of *R07B7.2* and thus increase the expression of the two transcripts, which consequently would decrease the expression of *R07B7.2[cd]*. Second, the insertion could cause a frameshift and insertion in the CDS of *R07B7.2[cd]*, which caused I474NL (ATA to AATTTA) and V471DL (GTA to GATTTA) in *R07B7.2[c]* and *R07B7.2[d]* (fig. 2*b*), respectively. These mutations might increase mRNA degradation. Taken together, our results demonstrated the effects of STR variation on gene expression and provided examples for potential underlying mechanisms.

### STR Variation Underlies Distant eQTL Hotspots

**Fig. 3.**
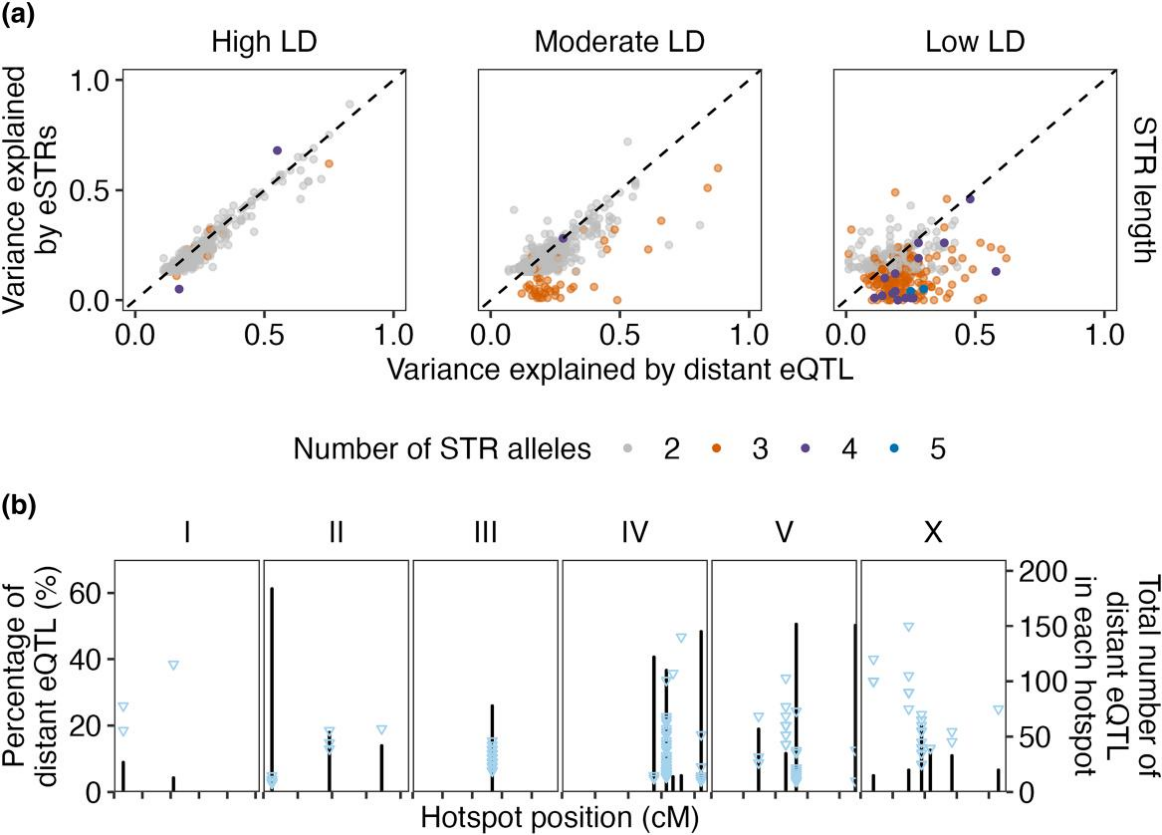
eSTRs underlying distant eQTL hotspots. (*a*) The VE by distant eQTL that were identified by GWA mapping experiments (Zhang, Roberto, et al. 2022) was plotted against the VE by the most significant eSTRs. Dots are colored by the number of STR alleles used in eSTR VE calculation. LD (*r*^2^) between eQTL and eSTRs were used to separate panels on the *x*-axis, with high LD (*r*^2^ ≥ 0.7), moderate LD (0.3 ≤ *r*^2^ < 0.7), and low LD (*r*^2^ < 0.3). The dashed lines on the diagonal are shown as visual guides to represent VE_eQTL_ = VE_eSTRs_. (*b*) The percentage of distant eQTL (*y*-axis on the left) that were associated with eSTRs in each distant eQTL hotspot (Zhang, Roberto, et al. 2022) across the genome (*x*-axis) is shown. Each triangle represents a common eSTR. Bar indicates the total number of distant eQTL (*y*-axis on the right) in each hotspot. Tick marks on the *x*-axis denote every 10 cM.

In addition to local eQTL, we also identified 3,360 distant eQTL for 2,553 transcripts from 2,382 genes (Zhang, Roberto, et al. 2022). Genetic variants underlying distant eQTL might affect genes encoding diffusible factors like TFs to regulate genes across the genome. After the identification of local eSTRs, we identified distant eSTRs that affect remote genes. Instead of testing all pSTRs across the genome for each transcript, we selected pSTRs that are within 2 Mb surrounding the quantitative trait locus (QTL) regions of interest for all distant eQTL of each transcript (supplementary fig. S1*a*, Supplementary Material online). We used LRT tests (as above, also see Materials and Methods) to associate pSTR length variation with expression variation. In total, 353,694 tests were performed for the effects of 2,743 pSTRs on the expression variation of 2,553 transcripts, each of which was tested for a median of 104 STRs (ranging from 1 to 1,005). We used the Bonferroni threshold (1.4E-7) to identify 1,857 distant eSTRs for 950 transcripts, with a median of three distant eSTRs (ranging from 1 to 127) (supplementary data S3, Supplementary Material online). We also compared the expression variation explained by each distant eQTL and the most significant distant eSTR, and the LD between them. Different from local eQTL-eSTR pairs (fig. 1*b*), most distant eQTL-eSTR pairs showed moderate (38%) or low (34%) LD (fig. 3*a*), suggesting a more independent role of distant eSTRs from distant eQTL in gene regulation than local eSTRs from local eQTL (figs. 1b and 3*a*). We have previously identified 46 distant eQTL hotspots that were enriched with distant eQTL (Zhang, Roberto, et al. 2022; fig. 3*b*). Genetic variants in these hotspots were associated with expression variation in up to 184 transcripts (Zhang, Roberto, et al. 2022). Here, we found 229 common distant eSTRs that were associated with at least five distant eQTL in each hotspot (fig. 3*b*). Common eSTRs might even underlie about half of all the distant eQTL in several hotspots (fig. 3*b*). Altogether, these results suggested the complementary regulatory effects of distant eSTRs to distant eQTL and hotspots.

We next investigated whether any of the common distant eSTRs were in genes encoding TFs or chromatin cofactors. We found nine TF genes and one chromatin cofactor gene that harbor common distant eSTRs (supplementary data S4, Supplementary Material online). For example, STR_12763 was a common eSTR for seven distant eQTL in the hotspot ranging from 26 to 27.5 cM on chromosome III (supplementary data S4, Supplementary Material online). STR_12763 is in the 3′ UTR of the TF gene, *atf-7* (Kudron et al. 2018), and overlaps with the binding sites of multiple miRNAs (supplementary fig. S6, Supplementary Material online). Variation in STR_12763 could affect the targeting of *atf-7* mRNAs by miRNAs to alter expression of the six transcripts (genes). However, none of the ten common distant eSTRs were also identified as local eSTRs for the genes in which they are located. So, we investigated whether any other common eSTRs, although not in known regulatory genes, were also identified as local eSTRs.

We found ten common distant eSTRs that were also local eSTRs for seven genes (supplementary data S4, Supplementary Material online). We previously mentioned STR_13795 (ATTTTT)_5_ as one of the two local eSTRs with enriched motif sequences. The variation of STR_13795 was associated with two transcripts of the gene, *cls-2*. Strains with STR contraction by about three repeats (17 bp) in STR_13795 showed significantly higher expression in both transcripts of *cls-2* than strains with the reference STR allele (supplementary fig. S7*a*, Supplementary Material online). Because STR_13795 was in the 3′ UTR of *cls-2*, the 17-bp deletion associated with expression of *cls-2* might affect the targeting by miRNAs (Sonenberg and Hinnebusch 2009; Jan et al. 2011). STR_13795 was also identified as a distant eSTR for another ten transcripts, including the gene *polq-1* (supplementary fig. S7*b*, Supplementary Material online). STR_13083 was identified as a local eSTR for *polq-1* and distant eSTRs for another nine transcripts, of which six had STR_13795 as an eSTR (supplementary figs. S7*b*and S8, Supplementary Material online). Most strains with length 30 and 13 in the STR_13795 also have length 16 and 15, respectively, in the STR_13083 (supplementary table S2, Supplementary Material online). Because STR_13795 was also associated with *polq-1*, STR_13795 was more likely to be the causal candidate than STR_13083 to alter the expression of the six overlapped target transcripts. The significant association between STR_13083 length variation and the expression variation of the six overlapped transcripts was identified because of the linkage between STR_13083 and STR_13795. The three transcripts that only had STR_13083 as their distant eSTRs could also be associated with the length variation of STR_13795, which was not tested for the three transcripts because it was too distant from the genes. Altogether, STR_13795 might affect the expression of all the 13 remote transcripts and genes by altering the expression of *cls-2* (supplementary fig. S7 and S8*b*, Supplementary Material online). We performed gene set enrichment analysis (GSEA) for the 13 genes on WormBase (Harris et al. 2020) and found significant enrichment in genes related to spindle and germline defectiveness (supplementary table S3, Supplementary Material online). The conserved protein, CLASP/CLS-2, is required for mitotic central spindle stability, oocyte meiotic spindle assembly, chromosome segregation, and polar body extrusion in *C. elegans* (Dumont et al. 2010; Espiritu et al. 2012; Maton et al. 2015; Pelisch et al. 2019; Schlientz and Bowerman 2020). To summarize, variation in STR_13795 might alter the expression of *cls-2*, which could further affect other related genes in the spindle assembly pathways.

### Oxidative Stress Potentially Drives STR Mutations

To explore the genome-wide influences of STRs on gene expression variation, we also wondered what factors might affect STR mutations and cause STR variation across *C. elegans*. DNA strand slippage during replication, DNA repair, and recombination processes can lead to STR mutations (Mirkin 2007). We reasoned that any genetic or environmental factors that are able to increase errors during these processes or decrease genome stability could increase STR mutation rates (Schmidt and Mitter 2004; Cooley et al. 2010). We hypothesized that if variation in genetic factors that affect genomic stability exists, the amount of total STR variation could be used as a quantitative trait for a GWA mapping study. We recently also developed a pipeline of mediation analysis to link gene expression variation to quantitative traits (Zhang, Roberto, et al. 2022). Thus, we sought to examine potential genetic and mediating factors underlying STR mutation variation.

**Fig. 4.**
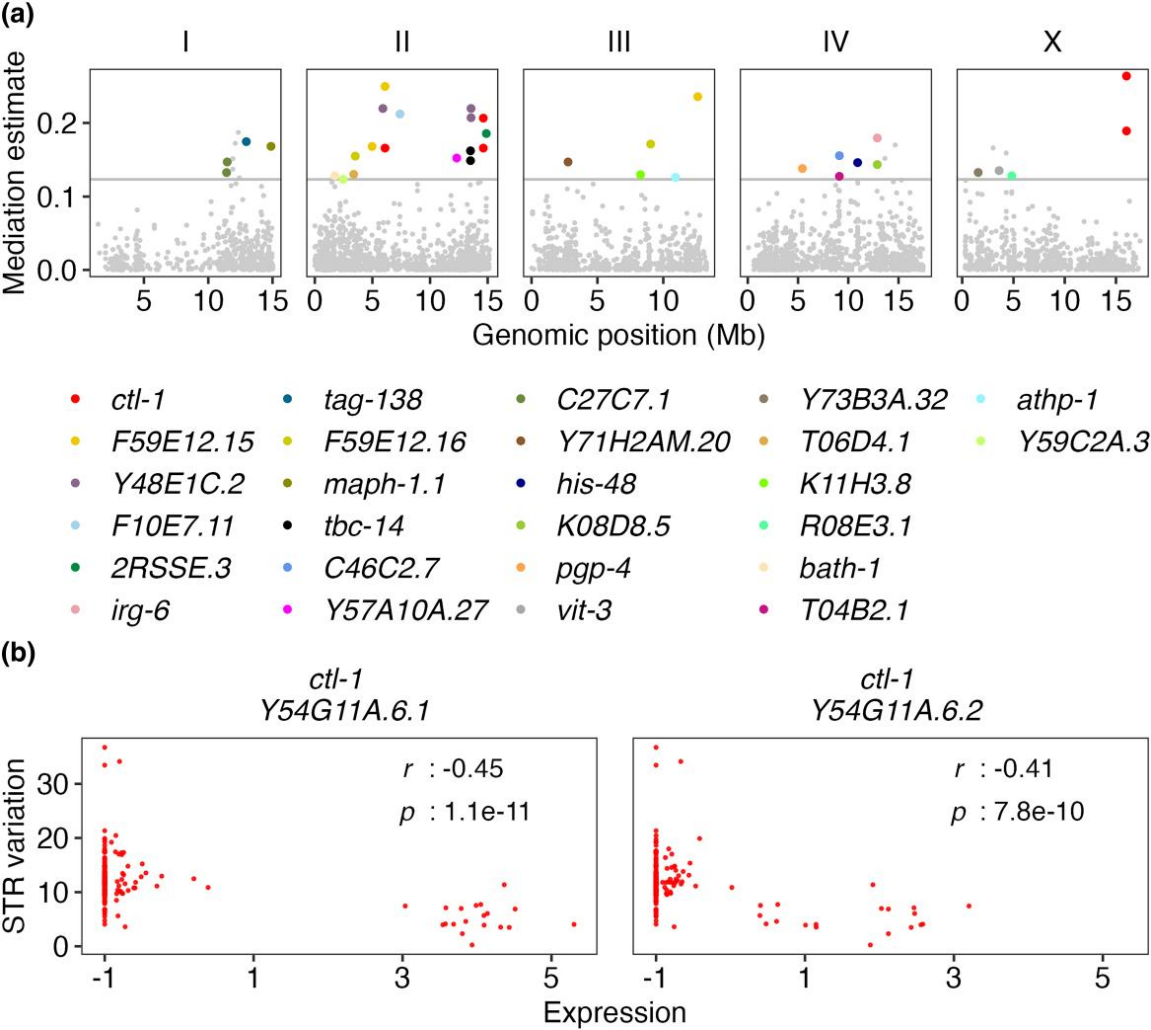
Mediation effects of *ctl-1* expression on STR variation. (*a*) Mediation estimates (*y*-axis) of transcript expression on STR variation are plotted against the genomic position (*x*-axis) of the eQTL. The horizontal gray line represents the 99th percentile of the distribution of mediation estimates. Mediator transcripts with adjusted *P* < 0.05 and interpretable mediation estimate greater than the 99th percentile estimates threshold are colored by their genes. Other tested mediator transcripts are colored gray. (*b*) The correlation of expression (*x*-axis) of two mediator transcripts to STR variation (*y*-axis) is shown. Each red dot represents a strain. The coefficient *r* and the *P*-value for each correlation using the two-sided Pearson's correlation tests are indicated in the top right.

We first defined an STR variation trait by counting reference and alternative STR alleles for each of the 207 strains in the 9,691 pSTRs (see Materials and Methods) (supplementary fig. S9*a*, Supplementary Material online). Deletions are the predominant mutations in STR mutations across wild *C. elegans* strains (supplementary fig. S9*a*, Supplementary Material online). We performed GWA mappings using two methods, leave-one-chromosome-out (LOCO) and INBRED (Widmayer et al. 2022), for this trait (see Materials and Methods). The INBRED method corrects more heavily for genetic stratification and many times decreases mapping power more than the LOCO method (Yang et al. 2011; Jiang et al. 2019; Widmayer et al. 2022). We detected six QTL with large QTL regions of interest on five of the six chromosomes using LOCO but no QTL using INBRED (supplementary fig. S9*b*and table S4, Supplementary Material online). We next used mediation analysis to link expression differences with total STR mutation variation (supplementary fig. S10, Supplementary Material online). Mediation analysis was performed for any transcripts with eQTL that overlap with the QTL regions of interest of the six QTL for STR variation, with the assumption that certain genetic loci affected the expression of certain genes, which subsequently affected STR variation (supplementary fig. S10, Supplementary Material online). We identified 31 significant mediator transcripts of 26 genes (fig. 4*a*). We performed GSEA for the 26 genes on WormBase (Harris et al. 2020) and found the most significant enrichment in genes related to transcription corepressor activity (supplementary table S5, Supplementary Material online). Transcription corepressors could alter chromatin structure (Harris et al. 2020) and subsequently affect STR variation. Moreover, the mediator gene, *ctl-1*, which showed the highest mediation estimate (fig. 4*a*), was found as a single enriched gene in four GO terms, such as “oxidoreductase activity acting on peroxide as acceptor” and “cellular oxidant detoxification” (supplementary table S5, Supplementary Material online). It had two transcripts, *Y54G11A.6.1* and *Y54G11A.6.2*, both of which were identified as significant mediators by multiple tests using different pairs of eQTL and QTL (fig. 4*a*). We found moderate negative correlations between the expression of the two *ctl-1* transcripts and STR mutation variation (fig. 4*b*), suggesting that the expression level of *ctl-1* might impact STR mutation variation. We regressed the STR variation trait by the expression of the transcript *Y54G11A.6.1* and performed GWA mappings. All the QTL mapped using the raw trait and LOCO disappeared in the mappings using the regressed trait (supplementary fig. S9*c*and table S4, Supplementary Material online), supporting that the expression variation of *ctl-1* might affect STR mutation variation. We also identified a new QTL at the position 14,625,147 bp on chromosome II in both LOCO and INBRED methods (supplementary fig. S9*c*and table S4, Supplementary Material online), suggesting that loci other than *ctl-1* might affect STR mutation variation as well.

The gene, *ctl-1*, encodes a cytosolic catalase in the detoxification pathway of reactive oxygen species (ROS) (Taub et al. 1999). Elevated expression of *ctl-1* and other antioxidant related genes, which likely enhanced resistance to oxidative stresses, were associated with lifespan elongation in *C. elegans* (Lin et al. 2019; Song et al. 2020). Oxidative damage can alter DNA secondary structure, affect genome stability and replication, and cause mutations (Poetsch 2020). Therefore, it is possible that the group of strains showing high levels of *ctl-1* expression managed to reduce STR mutations caused by oxidative damage over time and have lower levels of total STR mutations across the species (fig. 4*b*). We have previously detected five (one local and four distant) and six (one local and five distant) eQTL for expression variation of the two transcripts of *ctl-1*, *Y54G11A.6.1* and *Y54G11A.6.2*, respectively (Zhang, Roberto, et al. 2022). Among the 5,291 transcripts with detected eQTL, 4,430 transcripts had a single eQTL detected and only 30 transcripts were found with equal or more than 5 eQTL (Zhang, Roberto, et al. 2022). These results suggest that the expression of *ctl-1* was highly controlled and might be critical for adaptation to oxidative stresses.

**Fig. 5.**
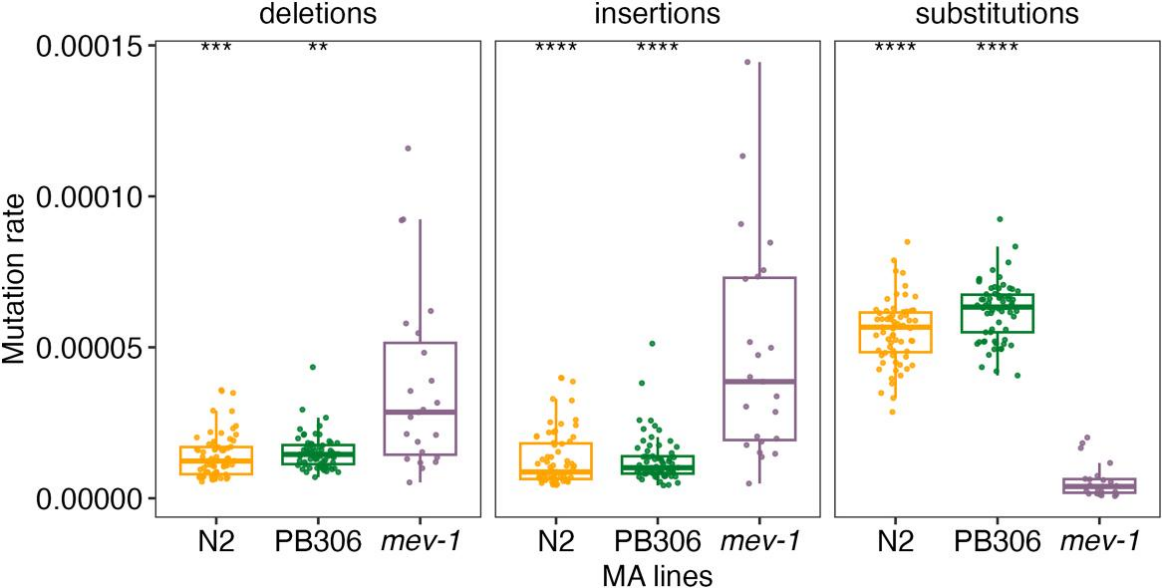
STR mutation rates in the MA lines. Comparison of STR mutation rates in deletions, insertions, and substitutions between the *mev-1* line and N2, and PB306 lines, respectively. Box edges denote the 25th and 75th quantiles of the data; and whiskers represent 1.5× the interquartile range. Statistical significance of difference comparisons (supplementary table S6, Supplementary Material online) was calculated using the two-sided Wilcoxon test and *P*-values were adjusted for multiple comparisons (Bonferroni method). Significance of each comparison is shown above each comparison pair (**adjusted *P ≤* 0.01; ***adjusted *P ≤* 0.001; ****adjusted *P ≤* 0.0001).

We further examined potential relationships between oxidative stresses and STR mutations using three MA line panels (Joyner-Matos et al. 2011; Matsuba et al. 2012; Saxena et al. 2019; Rajaei et al. 2021) that have undergone passage for many generations with minimal selection: 1) 67 MA lines that were derived from N2 and propagated for ∼250 generations; 2) 23 MA lines that were derived from a mutant strain, *mev-1* (with a missense mutation introgressed into N2, resulting in elevated oxidative stress), and propagated for ∼125 generations; and 3) 67 MA lines that were derived from PB306 (a wild strain) and propagated for ∼250 generations. We obtained raw sequencing data for these 157 MA lines and their three ancestors and called STR variation using the same method that we used for wild *C. elegans* strains (Zhang, Wang, et al. 2022) (see Materials and Methods). We calculated mutation rates for three different mutations (deletions, insertions, and substitutions) between the ancestor and each derived MA line and compared mutation rates across the three MA lines (fig. 5). We found that *mev-1* MA lines showed significantly higher mutation rates in deletions and insertions but significantly lower substitution rates than the other two MA lines (fig. 5 and supplementary table S6, Supplementary Material online). The significantly higher substitution rates of N2 and PB306 MA lines than the *mev-1* MA lines only existed in introns, 3′ UTRs, and intergenic regions, whereas the *mev-1* MA lines showed significantly higher mutation rates in deletions and insertions in different genomic features, including the CDS regions, promoters, and enhancers (supplementary fig. S11 and data S5, Supplementary Material online). The gene *mev-1* encodes a mitochondrial complex II SDHC (succinate dehydrogenase complex subunit C) (Ishii et al. 2013). The *mev-1* mutant was found to be highly sensitive to oxidative stress and showed reduced lifespan (Ishii et al. 2013). The high deletion and insertion rates in *mev-1* lines might be driven by their increased endogenous oxidative damage than the other two MA lines. Although the mutation rate of substitution was low in *mev-1* lines, deletions and insertions likely contributed most of the variation in STRs (supplementary fig. S9*a*, Supplementary Material online). The reduced lifespan (Ishii et al. 2013) of the *mev-1* MA lines might be associated with the high STR deletion and insertion rates in key gene regions, such as the CDS regions and promoters (supplementary fig. S11 and data S5, Supplementary Material online).

Altogether, these results suggest that oxidative stresses affect variation in STRs. Although a laboratory mutation in *mev-1* might have increased oxidative stresses and led to more deletions and insertions in STRs, natural genetic variation that promoted the expression of *ctl-1* might reduce oxidative stress, which might stabilize STRs to prevent mutations (fig. 4*b*).

## Discussion

Natural allelic variation in different classes of genomic loci contributes to gene expression variation (Albert et al. 2018; Fotsing et al. 2019; GTEx Consortium 2020; Jakubosky et al. 2020; Reinar et al. 2021; Zhang, Roberto, et al. 2022). We previously identified thousands of eQTL correlated with SNVs across wild *C. elegans* strains (Zhang, Roberto, et al. 2022). Here, we performed genome-wide analysis on how one of the most polymorphic and abundant repetitive elements, STRs (Zhang, Wang, et al. 2022), might affect expression variation in *C. elegans*. We identified nearly 5,000 associations between STR variation and expression variation of nearby and remote genes (figs. 1 and 3). It is important to note that the number of eSTRs that we detected only represents a conservative estimate because of the strict significance threshold that we applied. We selected STR genotypes and lengths with at least two common variants (frequency > 0.05) and modeled their effects on expression variation, respectively. Although genotypes and lengths were interchangeable in two-thirds of the pSTRs, the independent permutation tests using either type of data might have provided more robust inferences than pSTRs with noninterchangeable genotypes and lengths.

We previously performed GWA analysis on phenotypic variation in 11 organismal complex traits using pSTR length variation (Zhang, Wang, et al. 2022) and SNVs (Cook et al. 2016; Zdraljevic et al. 2017, 2019; Hahnel et al. 2018; Brady et al. 2019; Lee et al. 2019; Evans et al. 2020, 2021; Na et al. 2020; Zhang et al. 2021), respectively. Most of the significant STRs were located within or close to the QTL regions of interest identified using SNVs and GWA mappings, indicating close relationships between significant STRs and QTL. In the detection of eSTRs, we modeled pSTRs (Zhang, Wang, et al. 2022) within 2 Mb surrounding each of the 25,849 transcripts with reliable expression data (Zhang, Roberto, et al. 2022) (fig. 1). Close to 84% of transcripts found with local eSTRs were previously detected with local eQTL (Zhang, Roberto, et al. 2022), indicating close relationships between eSTRs and eQTL. Therefore, we further modeled pSTRs within 2 Mb surrounding the QTL regions of interest for transcripts with detected distant eQTL. Our results revealed important roles of distant eSTRs underlying distant eQTL and hotspots (fig. 3). Among transcripts with both eSTRs and eQTL, 48% of local and 28% of distant eSTR-eQTL pairs showed strong LD with each other and explained similar amounts of expression variance (figs. 1*b* and 3*a*). Future work using simulations and experiments is necessary to partition the contributions of eSTRs and eQTL to gene regulatory differences. Additionally, we also found that 17% of local and 34% of distant eSTR-eQTL pairs showed low LD with each other (figs. 1*b* and 3*a*). Among these low LD eSTR-eQTL pairs, 69% of local and 60% of distant eSTRs had three to six alleles used in LRT tests (figs. 1*b* and 3*a*), indicating independent roles of eSTRs, especially multiallelic STRs, in explaining expression variance. Note that the LD between eQTL and multiallelic STRs might be overestimated because we transformed multiallelic STR genotypes to biallelic to calculate LD (see Materials and Methods). Therefore, potentially more multiallelic eSTRs than we reported could have affected expression independently from eQTL. We further found eSTRs for 482 transcript expression traits (fig. 1*c*), which were not found with eQTL previously (Zhang, Roberto, et al. 2022). Many of these “unique” eSTRs explained more variance in the expression variation than the most significant SNVs nearby the transcripts (fig. 1*c*). With a combination of STR and SNV data, we showed improved estimation of narrow-sense heritability on the majority of transcript expression traits (supplementary fig. S3, Supplementary Material online). However, several confounding issues could have affected the results. Different methods, presumably with different mapping powers, were used to identify eQTL and eSTRs. STR data, which had higher frequencies in heterozygosity and multiallelic property than SNVs, were transformed before LD calculation. The estimation of VE by multiallelic eSTRs could be disturbed by its ineffective alleles. Meanwhile, differences in allele frequencies could also affect the estimation of VE.

STRs have been proposed to regulate gene expression using various molecular mechanisms (Gemayel et al. 2010; Raveh-Sadka et al. 2012; Afek et al. 2014; Conlon et al. 2016; Liu et al. 2018; Fotsing et al. 2019). We found local eSTR variants that caused a variety of mutations in the target transcripts. We dissected how a 3-bp insertion in an eSTR of the gene *R07B7.2* altered 3′ splice site to change alternative splicing efficiency and cause differential transcript usage (fig. 2). The function of the gene *R07B7.2* is not well understood but the expression of *R07B7.2* was found enriched in neurons, such as AVG and RIM (Harris et al. 2020). Future efforts could investigate the neural consequences of different transcript usage in the gene *R07B7.2*. Furthermore, we found that distant eSTRs might affect gene expression by disrupting miRNA binding in the 3′ UTRs of genes encoding TFs, such as ATF-7 (supplementary fig. S6, Supplementary Material online). Although the variation of STR_12763 and expression variation of *atf-7* were not significant in the local eSTR identification, it is possible that the effects of STR_12763 variation on the expression of *atf-7* were too small to be detected using data from 207 strains. But the small changes in the abundance of the ATF-7 protein might cause strong expression differences in the ATF-7 targets, which were detectable within the power of our study. In addition to TFs, we also identified that the eSTR STR_13795 might affect four genes (*cls-2*, *ddx-23*, *pck-2*, and *F54E7.9*) in the spindle assembly pathways through both local and distant regulation. It is most likely that *cls-2* is at the upstream of the pathway and its expression could affect the other three downstream genes. Several mutants of *cls-2* have been generated (Munoz et al. 2017). Future work could use these mutants to first examine whether the expression of *cls-2* affects the other three genes and then validate the role of STR_13795 mutations in expression regulation.

Not only did we observe eSTRs that altered gene expression, we also found that gene expression variation might affect STR mutations. We performed GWA mappings and mediation analysis on an STR variation trait and identified a candidate gene, *ctl-1*, that functions in the detoxification pathway of ROS (fig. 4, supplementary fig. S9*b*and table S5, Supplementary Material online). We observed low levels of genome-wide STR mutations in strains with high expression of *ctl-1* (fig. 4*b*), which might have increased the antioxidant capacity in the animal to stabilize the genome and reduce mutations. The effects of ROS on STR mutations were also revealed by *mev-1* MA lines, which experienced elevated oxidative stresses and showed higher STR deletion and insertion rates than wild type MA lines (fig. 5).

Not every strain with low levels of STR mutations had high levels of *ctl-1* expression (fig. 4*b*), suggesting STR mutations are polygenic. For example, other genes that are responsible for stress response in *C. elegans* might also affect STR mutations. Fungal infections were found to induce STR expansion in wheat (Schmidt and Mitter 2004). Various natural pathogens of *C. elegans* have been discovered (Troemel et al. 2008; Félix et al. 2011; Luallen et al. 2016; Zhang et al. 2016), and future work could compare STR mutations among *C. elegans* strains isolated from locations with or without known pathogens. Additionally, genes that are related to transcription corepressor activity (fig. 4*a* and supplementary table S5, Supplementary Material online) could also cause genome-wide effects on STR mutations.

Altogether, our study provides the first large-scale analysis of associations between STRs and gene expression variation in wild *C. elegans* strains. We highlighted the role of eSTRs in explaining expression variation and missing heritability. We also proposed that oxidative stress might have driven STR mutations globally. STRs have been proposed to facilitate adaptation and accelerate evolution (Vinces et al. 2009; Gemayel et al. 2010; King 2012; Gymrek et al. 2016; Press et al. 2018; Fotsing et al. 2019; Jakubosky et al. 2020; Reinar et al. 2021). Future work could use our data and analysis framework to study how STR variation affects complex traits and facilitates adaptation of *C. elegans* in the wild.

## Materials and Methods

### *C. elegans* Expression and STR Data

We obtained summarized expression data of 25,849 transcripts of 16,094 genes and genotypes of 9,691 pSTRs in 207 *C. elegans* strains from the original studies (Zhang, Roberto, et al. 2022; Zhang, Wang, et al. 2022). We also obtained 6,545 eQTL positions, their QTL regions of interest, and eQTL classification from the *C. elegans* eQTL study (Zhang, Roberto, et al. 2022).

### eSTRs Identification

#### STR Genotype Transformation

Genotypes of each pSTRs for each strain were transformed as previously described (Zhang, Wang, et al. 2022). Briefly, we used single digits (e.g., “0”, “1”, “2”) to represent STR genotypes in strains with homozygous alleles (e.g., “0|0”, “1|1”, “2|2”); we chose the smaller digits (e.g., “0”, “1”, “2”) to represent STR genotypes in strains with heterozygous alleles (e.g., “0|1”, “1|2”, “3|2”).

#### Selection of STRs for eSTRs Identification Tests

To identify local eSTRs, we selected pSTRs within 2 Mb surrounding each of the 25,849 transcripts with reliable expression measurements (Zhang, Roberto, et al. 2022). To identify distant eSTRs, we selected pSTRs within 2 Mb surrounding the QTL regions of interest for each of the 2,553 transcripts with detected distant eQTL (Zhang, Roberto, et al. 2022). Among selected pSTRs for each transcript, we further selected STRs with at least two common variants (frequency > 0.05) among strains with both STR genotype and expression data, and only retained strains with common STR variants.

#### LRT to Identify eSTRs

We treated STR genotypes as factorial variables and performed LRT on the full model *lm*(*expression* ∼ *STR*) and the reduced model *lm*(*expression* ∼ 1) using the *lrtest*() function in the R package *lmtest* (v0.9–39) (https://cran.r-project.org/web/packages/lmtest/index.html). The Bonferroni threshold was used to identify significant eSTRs. For each test using real data, we also performed another LRT using permuted data by shuffling STR genotypes across strains.

#### eSTR Identification Using STR Length Variation

Because different alleles of the same STR might have the same length and STR length variation might have stronger effect on gene expression than substitution, we performed LRT using the mean allele length of the two copies of each STR for each strain as factorial variables. We performed STR selection, permutation, LRT, and the Bonferroni threshold as above to identify eSTRs using STR length variation.

### LD and VE by eQTL and eSTRs

We calculated LD between top eSTRs and eQTL for transcripts with both regulatory sites detected. We used eQTL genotypes and STR genotypes to calculate LD for eSTRs detected by both STR genotype variation and STR length variation. Only strains used in eSTR identification were used for LD calculation. We acquired genotypes of wild strains at each eQTL from the hard-filtered isotype variant call format (VCF) file (CeNDR 20210121 release) (Cook et al. 2017). For processed STR genotypes, we further transformed all multiallelic variants into biallelic variants by converting all non-reference genotypes (1, 2, 3, etc.) to 1 and kept reference genotypes as 0. Then, we calculated LD correlation coefficient *r*^2^ for each STR–SNV and SNV–SNV pairs using the function *LD* () in the R package *genetics* (v1.3.8.1.2) (https://cran.r-project.org/package = genetics). LD was calculated as *r* = −*D*/*sqrt*(*p*(*A*) * *p*(*a*) * *p*(*B*) * *p*(*b*)), where *D* = *p*(*AB*) − *p*(*A*) * *p*(*B*). We also used untransformed STR genotypes and the generic function *cor*() (with Pearson correlation coefficient) in R to calculate the expression VE by each QTL and each top eSTR.

### Narrow-Sense Heritability Calculation

Narrow-sense heritability (*h*^2^) was calculated for each of the 25,849 traits using only SNVs or both SNVs and STRs. We extracted homozygous SNVs among the 207 strains from the same VCF used above and filtered out variants that had any missing genotype calls and variants that were below the 5% minor allele frequency using *BCFtools* (v.1.9) (Li 2011). We further pruned variants with a LD threshold of *r*^2^ ≥ 0.8 using *-indep-pairwise 50 10 0.8* in *PLINK* (v1.9) (Purcell et al. 2007; Chang et al. 2015) to generate an SNV genotype matrix containing 20,318 markers. For STRs, we first selected STRs with at least two common variants (frequency > 0.05) among the 207 strains in the transformed STR genotype data. Because STR missing calls were much more common than SNVs, we imputed the STR genotypes as previously described using the R package *missMDA* (v1.18) (Josse and Husson 2016; Zhang, Wang, et al. 2022) to generate an STR genotype matrix containing 3234 markers. Then, we combined both the SNV and the STR genotype matrixes to have an SNV–STR genotype matrix. We estimated *h*^2^ for each of the 25,849 traits using the SNV genotype matrix and the SNV–STR genotype matrix, respectively, and the functions *mmer*() and *pin*() in the R package *sommer* (v4.1.2) (Covarrubias-Pazaran 2016).

### DEU Analysis

We aligned RNA sequencing reads of the six replicates of the strains N2 and CB4856 using *STAR* (v2.7.5) (Dobin et al. 2013) to generate BAM files. We used the script *dexseq_prepare_annotation.py* in *DEXSeq* (v3.13) (Anders et al. 2012) and the General Feature Format file (WS276) from WormBase (Harris et al. 2020) to define exon counting bins, each of which represents one exon or part of an exon. Then, we used the script *dexseq_count.py* in *DEXSeq* to count the number of reads that overlap with each of the exon counting bins in the BAM files. Finally, we used the R function *testForDEU*() in *DEXSeq* to perform DEU analyses across all exon counting bins between the N2 and CB4856 strains. A total of 112,690 exon counting bins were defined in 13,355 genes across the genome (supplementary data S2, Supplementary Material online). Significance (*P*-values) in DEU analysis was adjusted by the Benjamini–Hochberg method and adjusted *P*-values ≤ 0.05 was used to identify significant DEU. A total of 976 significant DEU cases were found between N2 and CB4856 (supplementary data S2, Supplementary Material online).

### Genetic Basis of STR Variation

#### STR Variation Trait

We performed GWA mapping to identify the genetic basis of STR variation in *C. elegans*. For each of the 207 strains, we counted the total number (*N*_total_) of STRs with no missing genotypes among the 9,691 pSTRs and the total number of alternative alleles (*N*_alt_) for both copies at each site. The STR variation trait, which is used as the phenotypic input of GWA mappings, was calculated as *log_10_* (*N*_alt_/2*N*_total_).

#### GWA Mappings

We performed GWA mappings using the pipeline *Nemascan* (https://github.com/AndersenLab/NemaScan) as previously described (Widmayer et al. 2022). Briefly, we first generated an SNV genotype matrix as described above using the same VCF. Then, we used two approaches in the software *GCTA* (v1.93.2) (Yang et al. 2011; Jiang et al. 2019) to perform GWA mappings: 1) the LOCO approach, which uses the *-mlma-loco* function to both construct a kinship matrix using variants in all chromosomes except the chromosome in testing and perform the GWA mapping; and 2) the INBRED approach, which uses the *-maker-grm-inbred* function to construct a kinship matrix that is designated for inbred organisms and the *-fastGWA-lmm-exact* function for the GWA mapping (Yang et al. 2011; Jiang et al. 2019; Widmayer et al. 2022). An eigen-decomposition significance threshold (EIGEN) and a more stringent Bonferroni-corrected significance threshold (BF) were estimated in *Nemascan* for QTL identification. For EIGEN, we first estimated the number of independent tests (*N*_test_) within the genotype matrix using the R package *RSpectra* (v0.16.0) (https://github.com/yixuan/RSpectra) and *correlateR* (v0.1) (https://github.com/AEBilgrau/correlateR). EIGEN was calculated as −*log_10_*(0.05/*N*_test_). BF was calculated using all tested markers. Here, QTL were defined by at least one marker that was above BF. QTL regions of interest were determined by all markers that were above BF and within 1 kb of one another, and 150 more markers on each flank.

#### Mediation Analysis

We performed mediation analysis that is implemented in *Nemascan* to identify the mediation effect of gene expression on STR variation as previously described (Zhang, Roberto, et al. 2022). Briefly, for each QTL of STR variation, we used the genotype (*Exposure*) at the QTL, transcript expression traits (*Mediator*) that have eQTL (Zhang, Roberto, et al. 2022) overlapped with the QTL, and STR variation (*Outcome*) as input to perform mediation analysis using the *medTest*() function in the R package *MultiMed* (v2.6.0) (https://bioconductor.org/packages/release/bioc/html/MultiMed.html) and the *mediate*() function in the R package *mediation* (v4.5.0) (Tingley et al. 2014). Significant mediators were identified as those with adjusted *P* < 0.05 and interpretable mediation estimates greater than the 99th percentile of all estimates.

#### GWA Mapping for the Regressed STR Variation Trait

We regressed the STR variation trait by the expression of the transcript *Y54G11A.6.1* of the gene *ctl-1* and performed GWA mappings as described above.

### STR Variants in MA Lines

We obtained whole-genome sequence data in the FASTQ format of 160 MA lines, including N2 MA lines: the N2 ancestor and 67 MA lines; *mev-1* MA lines: the *mev-1* ancestor and 23 MA lines; and PB306 MA lines: the PB306 ancestor and 67 MA lines (NCBI Sequence Read Archive projects PRJNA395568, PRJNA429972, and PRJNA665851) (Saxena et al. 2019; Rajaei et al. 2021). We used the pipelines *trim-fq-nf* (https://github.com/AndersenLab/trim-fq-nf) and *alignment-nf* (https://github.com/AndersenLab/alignment-nf) to trim raw FASTQ files and generate BAM files for each line, respectively (Cook et al. 2017). We called STR variants for the 160 lines using the pipeline *wi-STRs* (https://github.com/AndersenLab/wi-STRs) (Zhang, Wang, et al. 2022).

### Mutation Rate of pSTRs in MA Lines

We calculated the STR mutation rate in MA lines as previously described (Zhang, Wang, et al. 2022) but using variant calls before filtering by 10% missing data. Briefly, between each MA line and its ancestor, we selected STR sites with reliable (“PASS”) calls in both lines. Then, for each STR, we compared the two alleles in the MA line to the two alleles in the ancestor, respectively, to identify insertion, deletion, substitution, or no mutation. The mutation rate (per-allele, per-STR, per-generation) *µ* for each type of mutation was calculated as *m*/2*nt* where *m* is the number of the mutation, *n* is the total number of reliable STRs, and *t* is the number of generations (Saxena et al. 2019; Rajaei et al. 2021).

### Statistical Analysis

Statistical significance of difference comparisons were calculated using the Wilcoxon test and *P*-values were adjusted for multiple comparisons (Bonferroni method) using the *compare_means*() function in the R package *ggpubr* (v0.2.4) (https://github.com/kassambara/ggpubr/). Enrichment analyses were performed using the one-sided Fisher's exact test and were corrected for multiple comparisons (Bonferroni method).

## Supplementary Material

msad067_Supplementary_Data

## Data Availability

The data sets and codes for generating all figures can be found at https://github.com/AndersenLab/Ce-eSTRs. ***Conflict of Interest Statement***. The authors declare no competing interests.
