# Supplementary material for: Interplay Between Polymorphic Short Tandem Repeats and Gene Expression Variation in Caenorhabditis elegans
Source: Mol Biol Evol. 2023 Mar 31;40(4):msad067. doi: 10.1093/molbev/msad067 (PMC10075192; doi:10.1093/molbev/msad067)
Supplement: msad067_Supplementary_Data [file msad067_supplementary_data.zip › eSTRs_supp_revision.pdf]

Supplementary Information

Interplay between polymorphic short tandem repeats and gene expression variation in *Caenorhabditis elegans*

Gaotian Zhang[1] and Erik C. Andersen[1,*]

1. Department of Molecular Biosciences, Northwestern University, Evanston, IL 60208, USA

[*]Corresponding author. E-mail: erik.andersen@gmail.com (E.C.A.)

Description of Additional Supplementary Files

File Name: Supplementary Data 1
Description: List of eSTRs for nearby genes.

File Name: Supplementary Data 2
Description: Differential exon usage analysis results

File Name: Supplementary Data 3
Description: List of eSTRs for remote genes.

File Name: Supplementary Data 4
Description: List of common distant eSTRs that are in genes encoding TFs or chromatin cofactors, or the distant eSTRs are also local eSTRs for the genes in which they are located.

File Name: Supplementary Data 5
Description: Comparison statistics of STR mutation rates in different genomic features among MA lines.

Supplementary Tables

Supplementary Table 1

Coverage from 12,057,474 to 12,057,485 bp on the chromosome V (ChrV) in six RNA sequencing BAM files generated by *Kallisto* (Bray et al. 2016) is shown for the replicates of two *C. elegans* strains N2 and CB4856. Reads mapped to multiple genes were removed.

Position on ChrV (bp)	CB4856 GZ_B1_H3	CB4856 GZ_B5_I9	CB4856 GZ_B6_C6	N2 GZ_B7_A7	N2 GZm_B6_H3	N2 GZr_B6_E4
12057474	196	112	252	310	225	195
12057475	197	114	252	312	223	196
12057476	197	114	252	312	223	196
12057477	198	117	252	312	223	199
12057478	187	111	252	306	224	192
12057479	188	111	252	306	225	193
12057480	19	9	28	157	122	101
12057481	18	9	28	158	122	103
12057482	14	9	28	158	120	104
12057483	14	9	28	158	119	106
12057484	14	9	28	158	119	107
12057485	14	9	26	158	119	106

Supplementary Table 2

Number of strains with reference (REF) or alternative (ALT) allele lengths in STR_13795 and STR_13083. Only 186 strains with expression data and genotypes at both STR sites are included.

STR			STR_13795	
	Allele		REF	ALT
		Length	30	13
STR_13083	REF	16	133 strains	15 strains
	ALT	15	6 strains	32 strains

Supplementary Table 3

GSEA results of 13 genes that were associated with STR_13795 in the gene *cls-2*.

Enrichment term	Expected count	Observed count	Enrichment Fold Change	*p* value	Adjusted *p* value	Enriched gene
Oocytes disorganized	0.13	2	16	0.0003	0.072	*ddx-23, F37C12.1*
Tumorous germline	0.16	2	13	0.00054	0.072	*ddx-23, F37C12.1*
spindle orientation variant	0.18	2	11	0.0008	0.072	*ddx-23, F54E7.9*
spindle defective early embryo	0.42	3	7.1	0.0009	0.072	*ddx-23, F54E7.9, pck-2*
microtubule organization biogenesis variant	0.5	3	6	0.0016	0.079	*ddx-23, F54E7.9, pck-2*

Supplementary Table 4
GWA QTL and regions of interest for the raw and regressed STR variation traits.

Trait	GWA method	QTL chromosome	QTL peak position	Start position of QTL region of interest	End position of QTL region of interest
STR variation	LOCO	I	12153609	4754801	13671576
STR variation	LOCO	II	2706647	1477894	15272855
STR variation	LOCO	III	4892173	2807201	13718059
STR variation	LOCO	IV	13760657	2278611	15378958
STR variation	LOCO	X	2603542	826476	8910667
STR variation	LOCO	X	14551237	14217837	17696299
Regressed STR variation	LOCO	II	11566198	10505390	11842443
Regressed STR variation	LOCO	II	14625147	13968708	15272855
Regressed STR variation	INBRED	II	14625147	13968708	15272855

Supplementary Table 5
GSEA results for the 26 significant mediator genes for the STR variation trait.

Enrichment term	Expected count	Observed count	Enrichment Fold Change	*p* value	Adjusted *p* value	Enriched gene
Transcription corepressor activity	0.046	2	43	1.4E-05	0.0045	*athp-1, F10E7.11*
Negative regulation of transcription by RNA polymerase II	0.14	2	15	0.00037	0.058	*athp-1, F10E7.11*
Actin binding	0.15	2	13	0.00047	0.058	*maph-1.1, tag-138*
Oxidoreductase activity acting on peroxide as acceptor	0.039	1	26	0.00072	0.058	*ctl-1*
Peptidyl-threonine phosphorylation	0.047	1	21	0.0011	0.066	*F59E12.15*
Cellular oxidant detoxification	0.059	1	17	0.0016	0.086	*ctl-1*
ABC-type transporter activity	0.062	1	16	0.0018	0.086	*pgp-4*
Detoxification	0.068	1	15	0.0021	0.086	*ctl-1*
Cellular response to toxic substance	0.071	1	14	0.0024	0.086	*ctl-1*
Regulation of GTPase activity	0.078	1	13	0.0028	0.089	*tbc-14*

Supplementary Table 6

Comparison of mutation rates among MA lines using two-sided Wilcoxon tests and Bonferroni method for multiple testing correction.

Mutation	group1	group2	*p* value	Adjusted *p* value
deletions	*mev-1*	N2	2.46E-05	0.00015
deletions	*mev-1*	PB306	1.79E-04	0.0011
insertions	*mev-1*	N2	1.56E-07	9.4E-07
insertions	*mev-1*	PB306	1.67E-08	1E-07
substitutions	*mev-1*	N2	1.06E-12	6.3E-12
substitutions	*mev-1*	PB306	1.06E-12	6.3E-12

Supplementary Figures

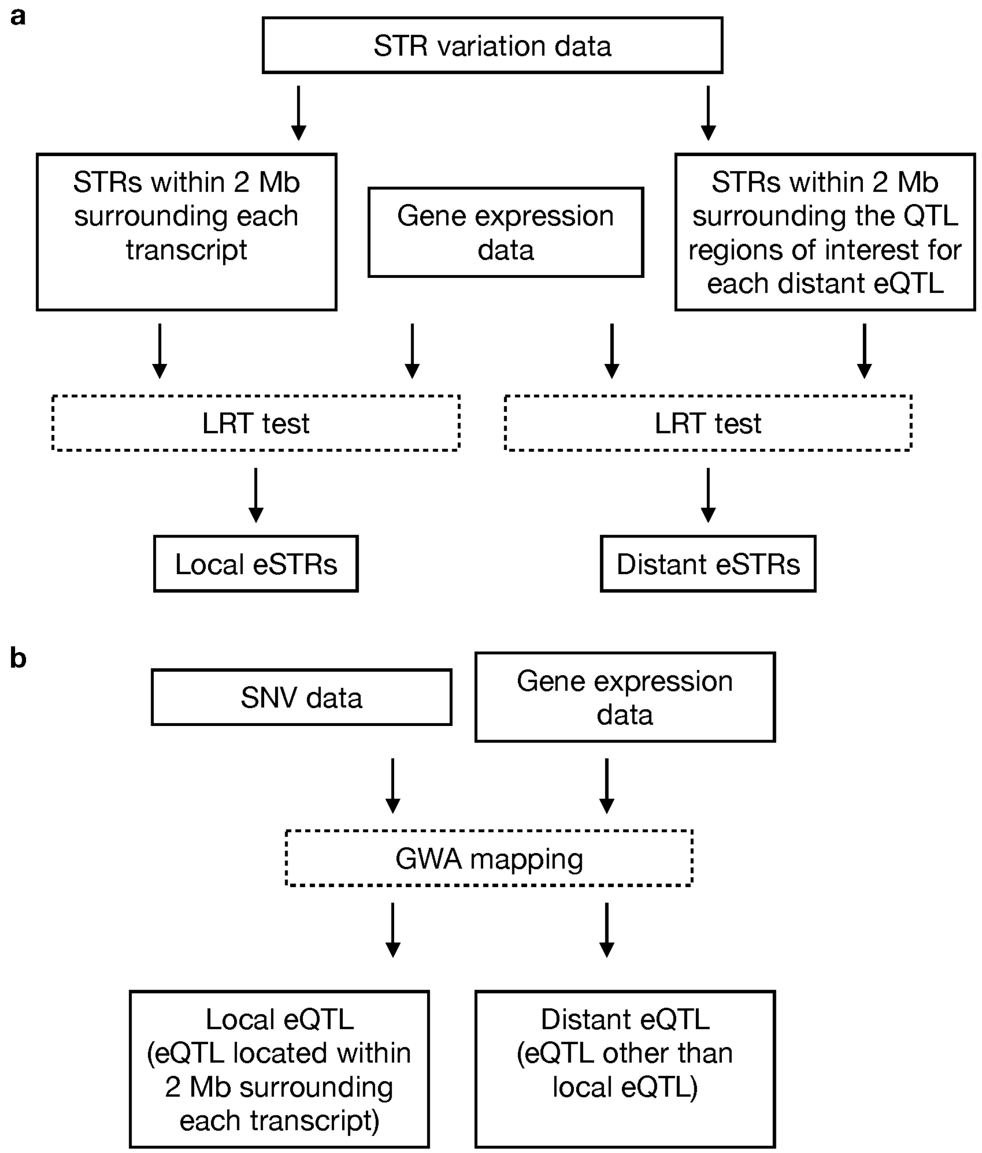

Supplementary Fig. 1
Illustration of eQTL and eSTRs analyses. a. Illustration of eSTR analysis and definitions of local and distant eSTRs. **b**. Illustration of eQTL analysis and definitions of local and distant eQTL (Zhang et al. 2022).

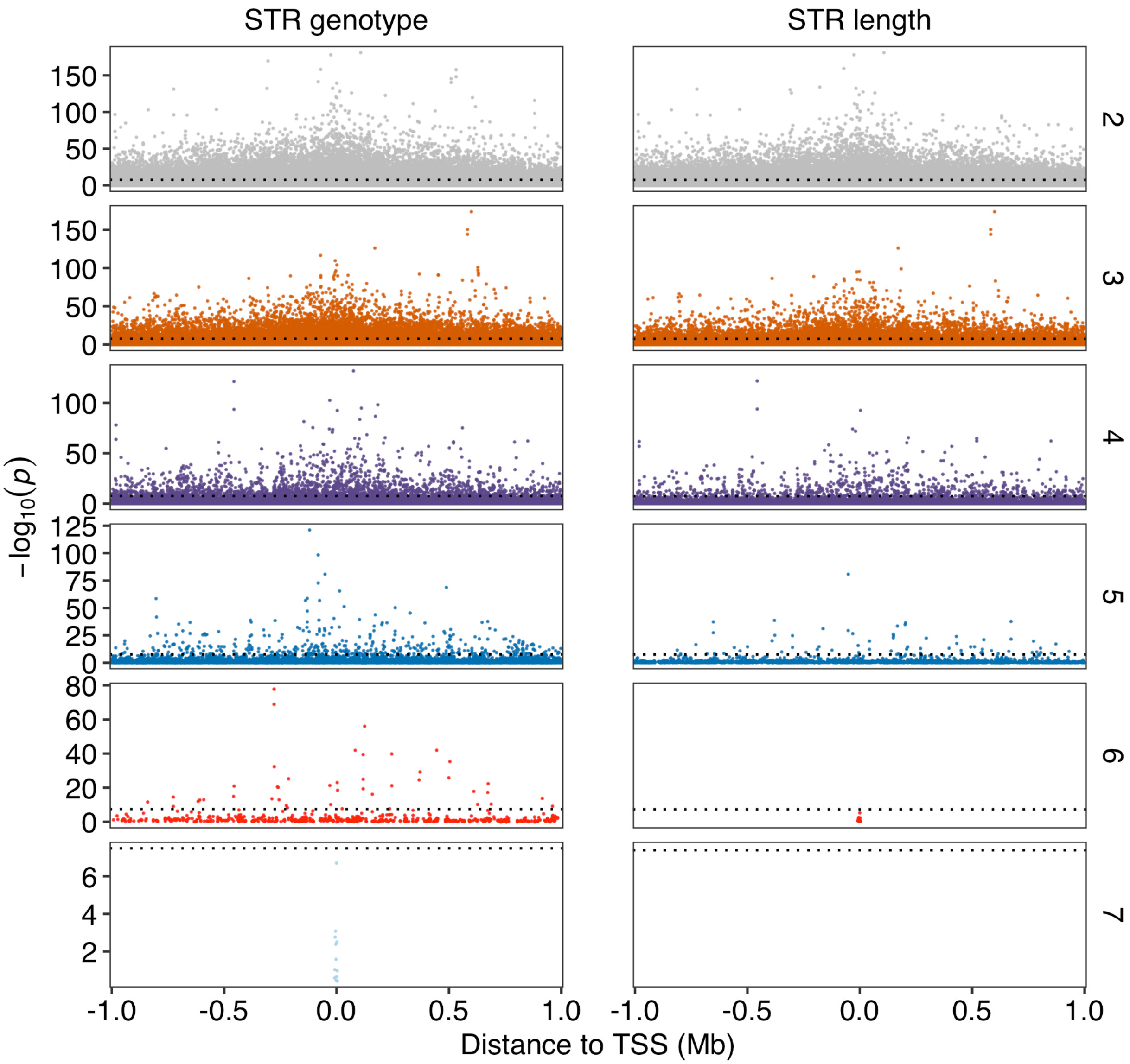

Supplementary Fig. 2

Identification of expression STRs (eSTRs) using Likelihood-Ratio Tests (LRT) on full (including STR variation as a variable) and reduced (excluding STR variation as a variable) models. The effects of STR variation in genotype (left panel) or length (right panel) were analyzed separately as factorial variables. Each dot represents a test between real STR data and transcript expression variation and is plotted with the distance of the STR to the transcription start site (TSS) of the transcript (x-axis) against its -log10 (*p*) value (y-axis on the left). The black dotted horizontal lines represent Bonferroni thresholds. The six panels on the y-axis (on the right) represent STRs with different numbers of alleles.

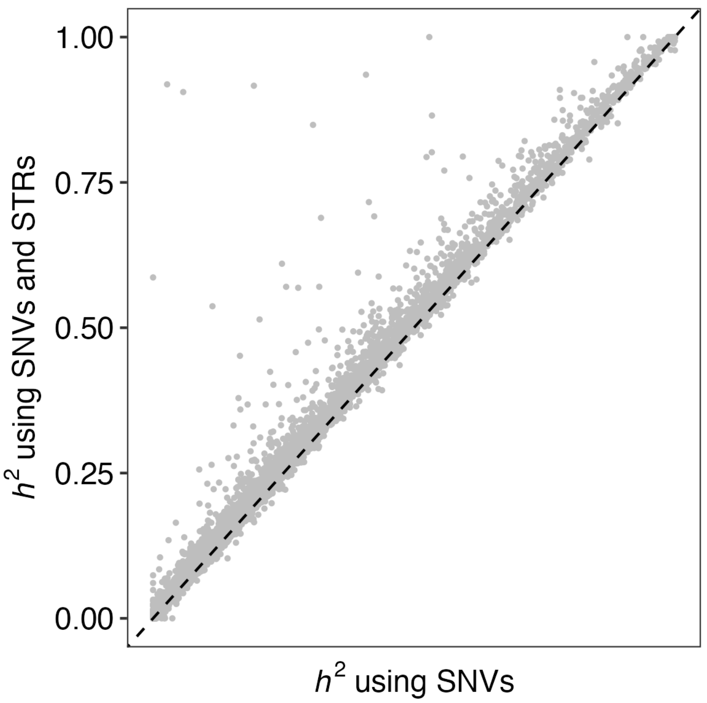

Supplementary Fig. 3

Narrow-sense heritability (h^2) for 25,849 transcript expression traits using only SNVs (x-axis) or both SNVs and STRs (y-axis). The dashed line on the diagonal is shown as a visual guide to represent equal values.

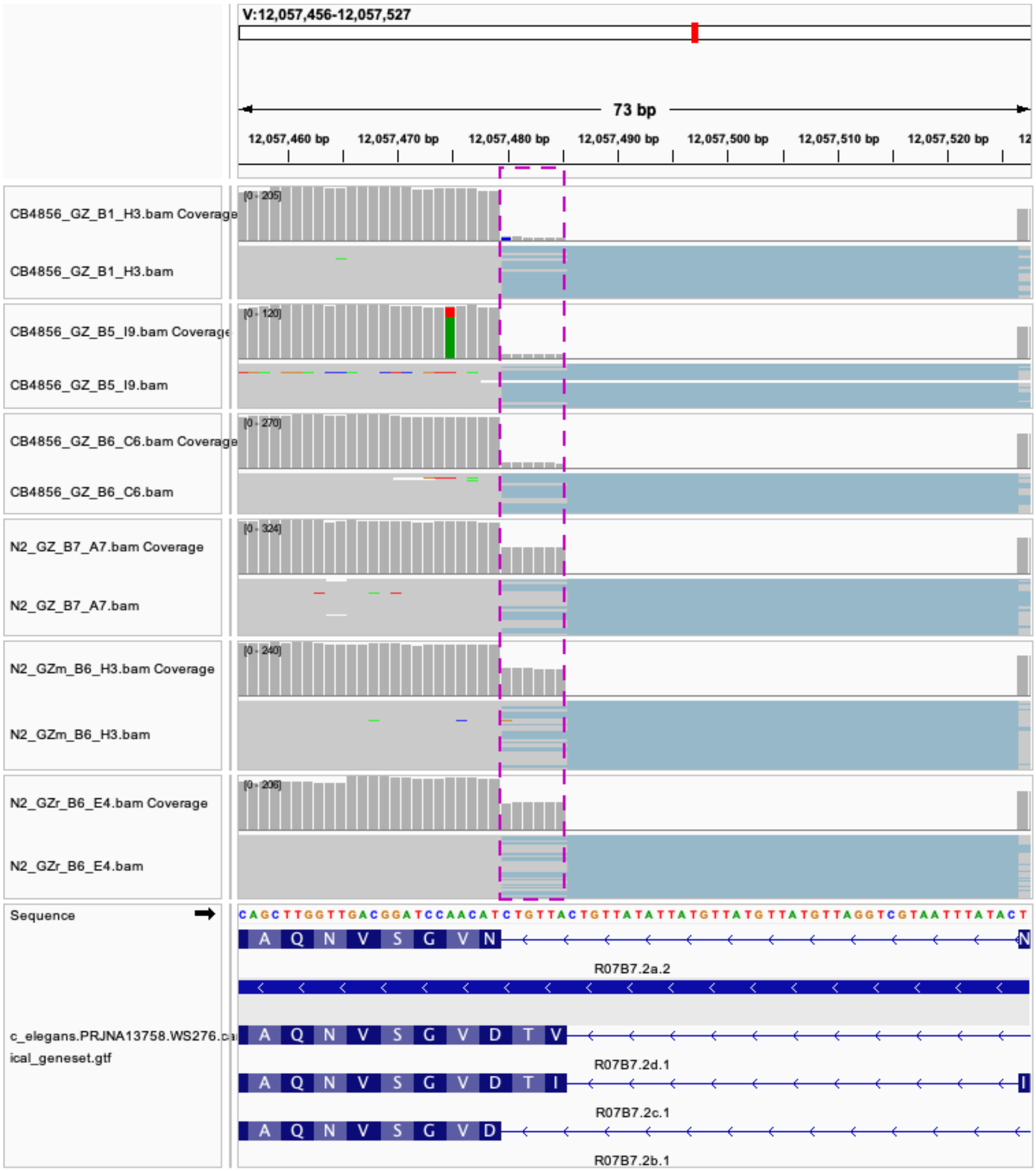

Supplementary Fig. 4

The coverage at a splicing junction of the gene *R07B7.2*. A screenshot of IGV (Robinson et al. 2011) is shown, including six RNA sequencing BAM files generated by Kallisto (Bray et al. 2016) for replicates of two *C. elegans* strains CB4856 and N2. BAM files names are indicated on the left. Gene structures of four *R07B7.2* transcripts are shown at the bottom. The purple dashed rectangle indicates the genomic region from 12,057,480 to 12,057,485 bp on the chromosome V. The gene *R07B7.2* is oriented in the 3′–5′ direction. Reads mapped to multiple genes were removed.

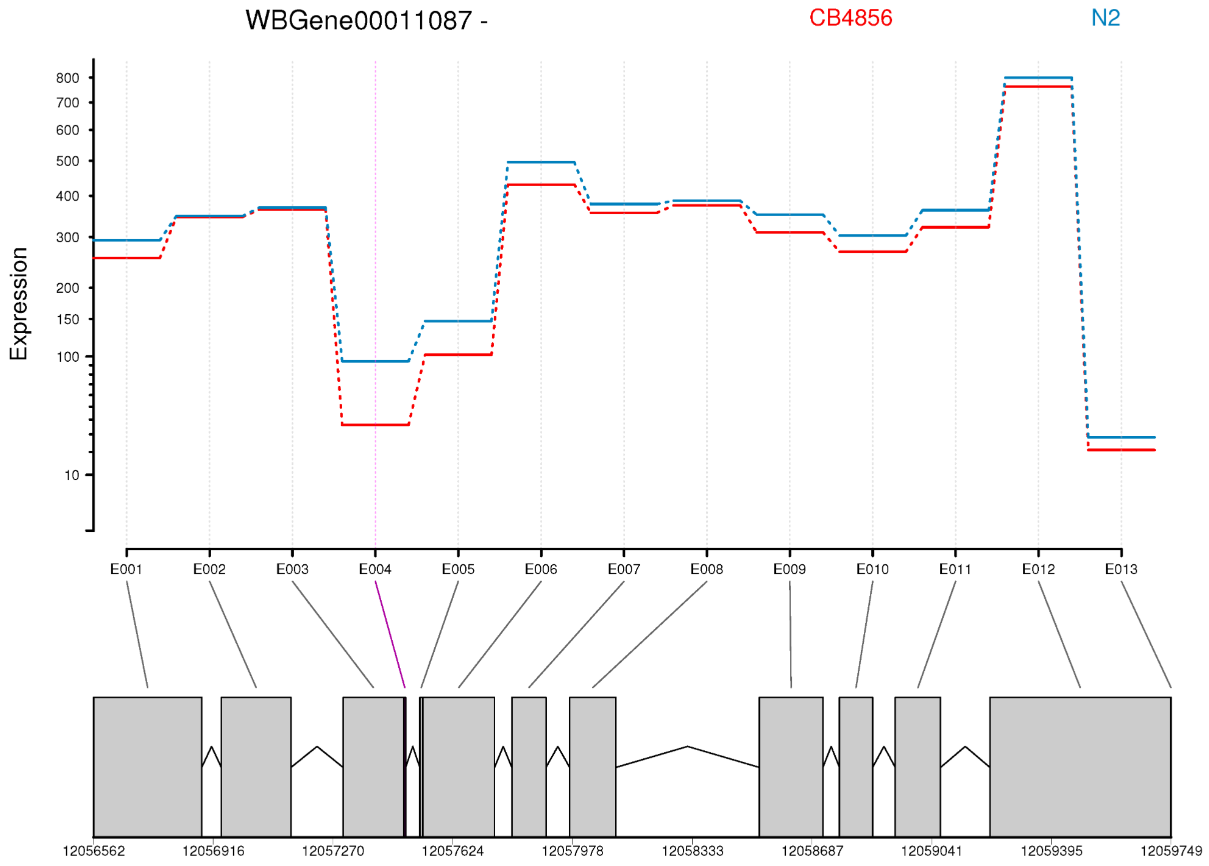

Supplementary Fig. 5

Differential exon usage in the gene *R07B7.2* between the *C. elegans* strains CB4856 and N2. Expression of different exon counting bins (Supplementary Data S2) in the gene *R07B7.2* (WormBase gene ID: WBGene00011087) are shown. The structures of the 13 exon counting bins in the gene *R07B7.2* are shown at the bottom. The pink vertical dashed line indicates significant differential usage (adjusted *p*-value: 6.8E-10) at the exon bin E004 between the CB4856 and N2 strains. E004 is the genomic region from 12,057,480 to 12,057,485 bp on the chromosome V (Supplementary Data S2). The plot was generated by the function *plotDEXSeq()* in *DEXSeq* (v3.13) (Anders et al. 2012).

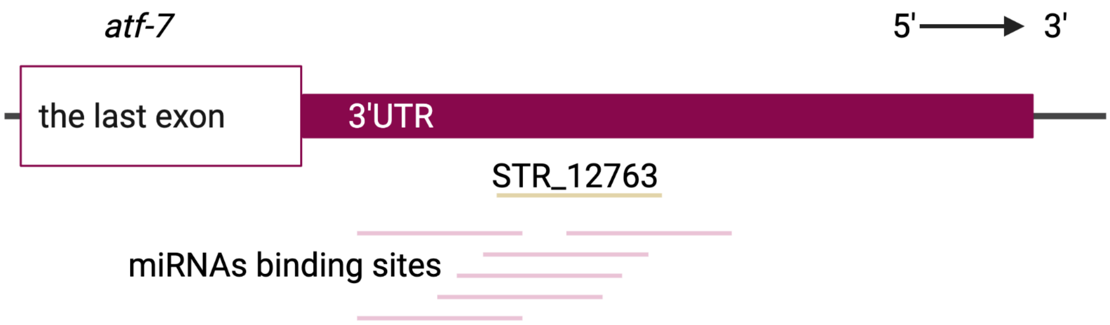

Supplementary Fig. 6
STR_12763 in 3'UTR of the TF gene, *atf-7*, might affect miRNA binding sites. Graphic illustration of the 3'UTR of *atf-7*, the STR_12763 (the light brown line), and predicted binding sites of miRNAs (pink lines) based on WormBase (Harris et al. 2020). Created using BioRender.com.

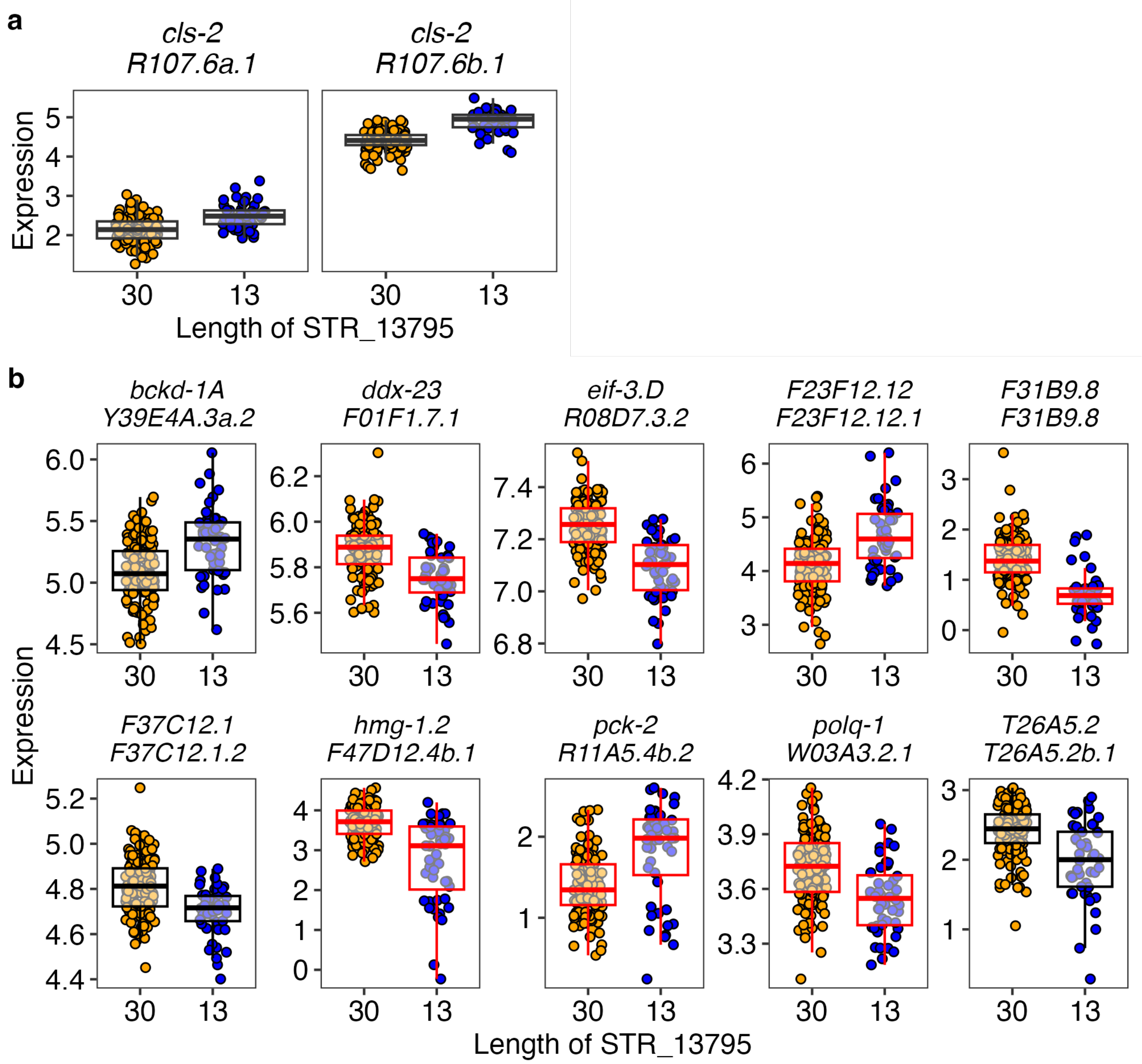

Supplementary Fig. 7

The local and distant eSTR, STR_13795. STR_13795 was identified as local eSTRs for two transcripts of the gene *cls-2* **a** and distant eSTRs for ten other transcripts **b**. Tukey box plots showing expression variation of the 12 transcripts between strains with different lengths of the STR_13795 are shown and colored red for those transcripts with STR_13083 as an eSTR (Supplementary Fig. 8). Each point corresponds to a strain and is colored orange and blue for strains with the N2 reference allele and the alternative allele, respectively. Box edges denote the 25th and 75th quantiles of the data, and whiskers represent 1.5× the interquartile range.

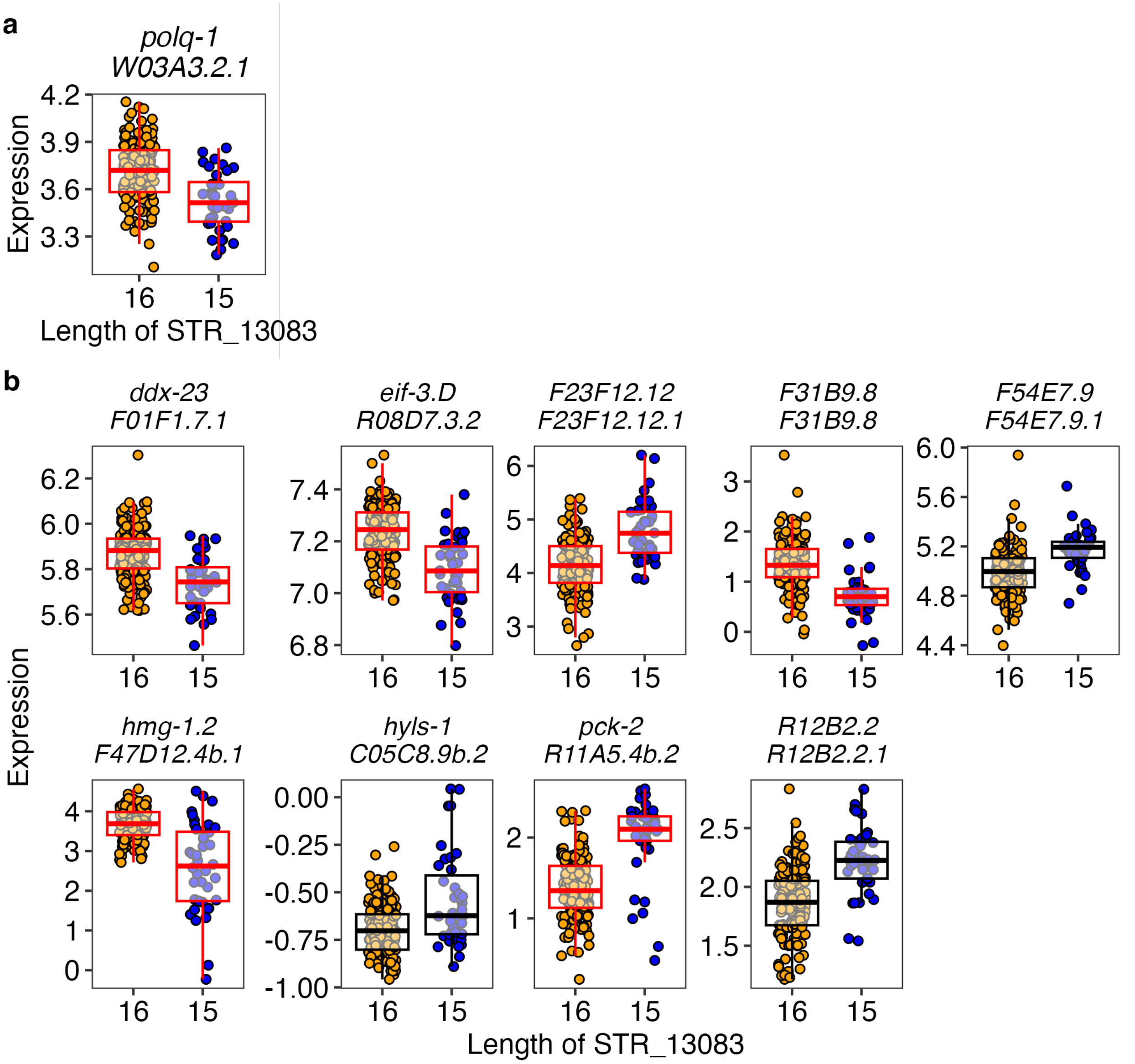

Supplementary Fig. 8

The local and distant eSTR, STR_13083. STR_13083 was identified as local eSTRs for the transcript of the gene *polq-1* **a** and distant eSTRs for nine other transcripts **b**. Tukey box plots showing expression variation of the ten transcripts between strains with different lengths of the STR_13083 are shown and colored red for those transcripts with STR_13795 as an eSTR (Supplementary Fig. 7). Each point corresponds to a strain and is colored orange and blue for strains with the N2 reference allele and the alternative allele, respectively. Box edges denote the 25th and 75th quantiles of the data; and whiskers represent 1.5× the interquartile range.

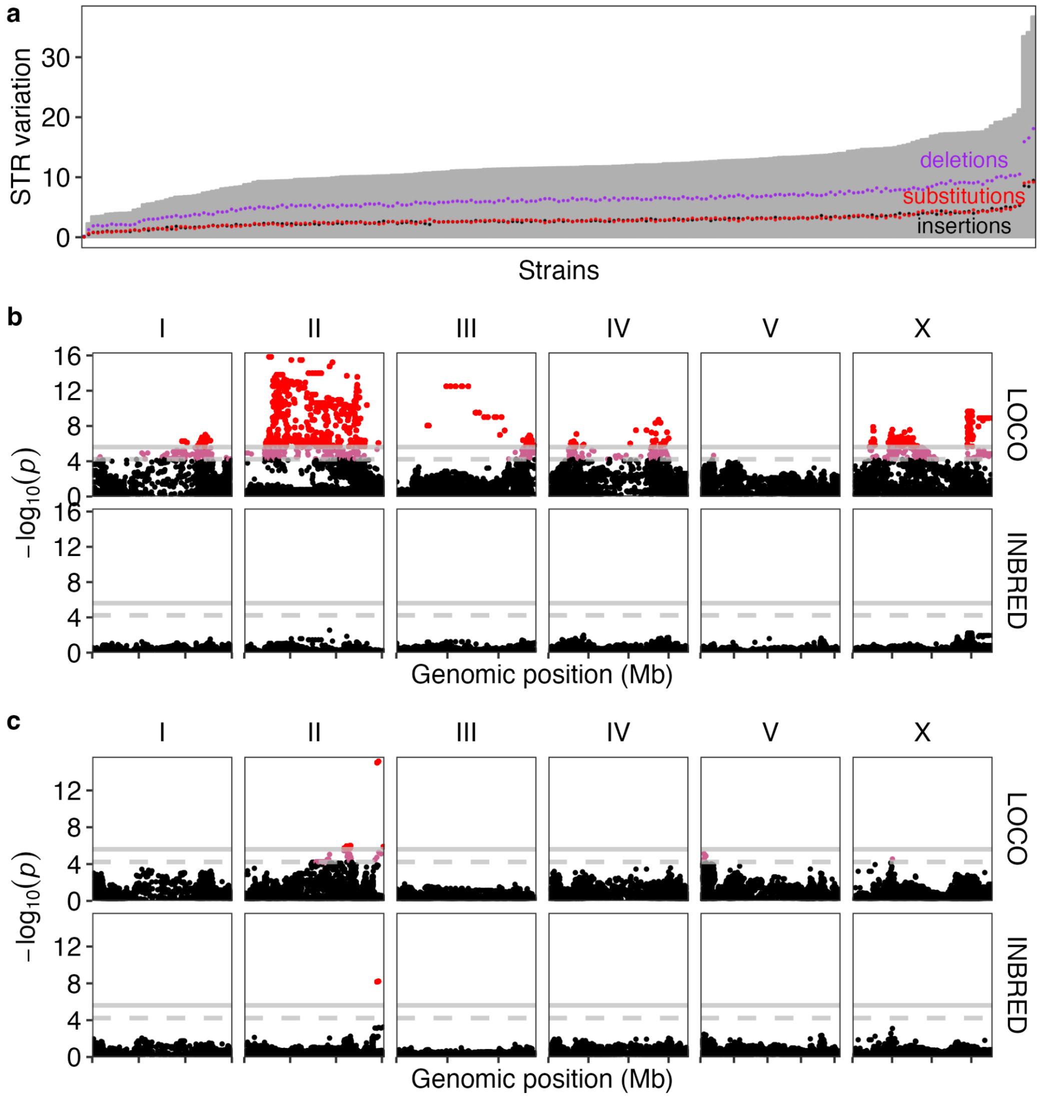

Supplementary Fig. 9

Genetic basis underlying STR variation. a The distribution of an STR variation trait across 207 strains is shown as gray bars. The STR variation traits calculated by deletions, insertions, and substitutions for each strain are shown as dots and colored purple, black, and red, respectively. **b** Manhattan plots indicating the GWA mapping results for STR variation across 207 strains using LOCO and INBRED approaches are shown, respectively. **c** Manhattan plots indicating the GWA mapping results for STR variation regressed by the expression of *Y54G11A.6.1* of the gene *ctl-1* across 206 strains using LOCO and INBRED approaches are shown, respectively. In **b** and **c**, each point represents an SNV that is plotted with its genomic position (x-axis) against its -log10(*p*) value (y-axis) in mapping. SNVs that pass the genome-wide EIGEN threshold (the dashed gray horizontal line) and the genome-wide Bonferroni threshold (the solid gray horizontal line) are colored pink and red, respectively. QTL were identified using the Bonferroni threshold. The tick marks on the x-axis denote every 5 Mb.

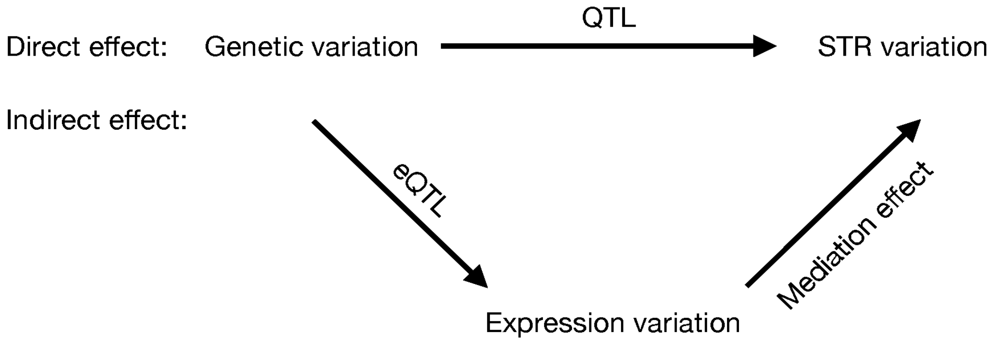

Supplementary Fig. 10

Illustration of the mediation analysis. Genetic variation could affect STR variation directly using QTL or indirectly to first cause expression variation using eQTL and then affect STR variation through mediation effect of gene expression.

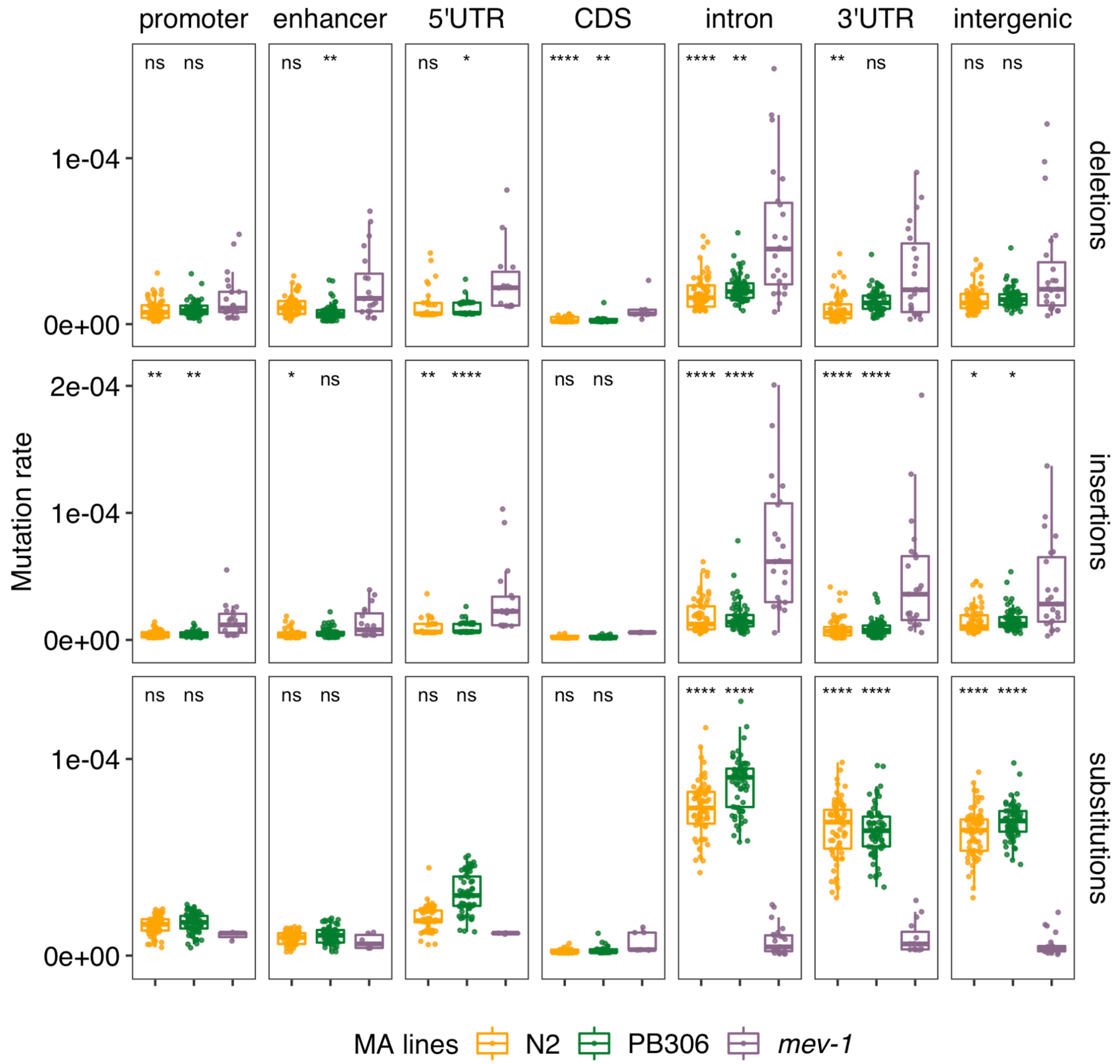

Supplementary Fig. 11

Comparison of STR mutation rates in deletions, insertions, and substitutions between the *mev-1* line (purple) and N2 (orange), and PB306 (green) lines, respectively, in different genomic features. Box edges denote the 25th and 75th quantiles of the data; and whiskers represent 1.5× the interquartile range. Statistical significance of difference comparisons (supplementary file S2) was calculated using the two-sided Wilcoxon test, and *p*-values were adjusted for multiple comparisons (Bonferroni method). Significance of each comparison is shown above each comparison pair (*: adjusted $p \leq 0.05$; **: adjusted $p \leq 0.01$; ***: adjusted $p \leq 0.001$; ****: adjusted $p \leq 0.0001$).

REFERENCES

Anders S, Reyes A, Huber W. 2012. Detecting differential usage of exons from RNA-seq data. *Genome Res.* 22:2008–2017.

Bray NL, Pimentel H, Melsted P, Pachter L. 2016. Near-optimal probabilistic RNA-seq quantification. *Nat. Biotechnol.* 34:525–527.

Harris TW, Arnaboldi V, Cain S, Chan J, Chen WJ, Cho J, Davis P, Gao S, Grove CA, Kishore R, et al. 2020. WormBase: a modern Model Organism Information Resource. *Nucleic Acids Res.* 48:D762–D767.

Robinson JT, Thorvaldsdóttir H, Winckler W, Guttman M, Lander ES, Getz G, Mesirov JP. 2011. Integrative genomics viewer. *Nat. Biotechnol.* 29:24–26.

Zhang G, Roberto NM, Lee D, Hahnel SR, Andersen EC. 2022. The impact of species-wide gene expression variation on Caenorhabditis elegans complex traits. *Nat. Commun.* 13:1–13.
